# Human-elephant conflicts and attitude of the local communities toward African elephant (*Loxodonta africana*) conservation in Kafta Sheraro National Park, Tigray region, Ethiopia

**DOI:** 10.7717/peerj.19428

**Published:** 2025-05-22

**Authors:** Fitsum Temesgen, Bikila warkineh

**Affiliations:** 1 Center for Environmental Sciences (Environmental Resources Conservation and Management), Addis Ababa University, Addis Ababa, Tigray, Ethiopia; 2 Plant Biology and Biodiversity Management and Environmental Sciences, Addis Ababa University, Addis Ababa, Ethiopia

**Keywords:** Human-elephant conflict, Community attitude, Wildlife conservation, Kafta Sheraro National Park

## Abstract

Human-wildlife conflict (HWC), particularly elephant crop raiding, has been increasing over the past decade in Kafta Sheraro National Park (KSNP). The objectives of this study were to assess the degree of KSNP natural resources utilization by the local community, the existing human-elephant conflict (HEC), trends of the conflict, methods used to minimize their negative impacts, and community attitudes and socio-demographic influencing factors regarding the conservation of African elephant in rather than and KSNP. The survey was carried out from November 2018 to September 2020. A total of 395 household heads were selected systematically from seven kebeles (the lowest governmental administrative units of Ethiopia). Direct field observations, household-based questionnaire surveys, focus group discussions, and key informant interviews (*i.e.*, administrators, professional experts, and park management staffs) were applied. The majority (74.51%) of the local communities utilized the park resources as grazing for livestock followed by fuel wood sources (46.04%), water sources (39.57%), and house construction materials (30.38%). More than 72% of the respondents suggested that crop raiding by elephants is a serious problem in the study area and increased in the past ten years. About 60.9% and 60.51% of the respondents mentioned that elephant-induced crop damage was during the wet season and at night, respectively. Crop damage was relatively high as cropland found inside and at the periphery of the park. Majorities of the respondents recommended that gun sounds/banging noisy materials (81.99%) and lighting fire/flashlight (44.95%) were the most common traditional protection methods from elephant crop damage. More than 56% of the respondents had positive attitudes toward the conservation of KSNP and elephant. About 54.18% of respondents were aware of park conservation and their awareness varied with age, education level, gender, settlement condition, and distance between settlement and park. The probability that males tended to respond to awareness was 3.5 times higher than that of female respondents. Males were more likely to have awareness about the aims of KSNP conservation and related issues. Factors influencing the attitude of the local community toward KSNP and elephant conservation in the area were age, education level, the distance between settlement and park, and their awareness status. Trends and levels of crop damage by elephants negatively influenced communities’ attitudes toward elephant conservation. Significant variation (*P* < 0.05) of respondents was observed on HEC, trends of crop damage, mitigation measures, awareness, and their attitudes toward protected area (PA) and elephant conservation. Therefore, the smooth coexistence of KSNP and wildlife/elephants with the local communities could be maintained by establishing buffer zones in the area to ensure conservation sustainability and community livelihoods.

## Introduction

Human-wildlife conflicts (HWC) often occur in human settlement-dominated areas (Anand & Radhakrishna, 2017; Acharya et al., 2016) and when wildlife and humans overlap and share the same resources (Lamarque et al., 2009). HWC can be defined as the negative interactions between humans and wildlife (Jacobsen & Linnell, 2016; Draheim et al., 2015; Messmer, 2009). It has recently become a fundamental aspect of wildlife management (Fernando et al., 2005). The conflict between conservation principles and other human interests is to be considered when analyzing and addressing human-wildlife interactions (Redpath, Bhatia & Young, 2014). HWC had been widely increasing across the world and is one of the main obstacles to wildlife conservation (Redpath et al., 2013) and it exists in different forms as humans continue to encroach on wildlife habitats (Lamarque et al., 2009). In Africa, the negative interactions between humans and wildlife have been recognized a global conservation issue (Woodroffe, Thirgood & Rabinowitz, 2005).

The primary causes of HWC include land use change, climate change and variability, and habitat degradation (Mukeka et al., 2019; Lamarque et al., 2009; Wittemyer et al., 2008; Hoare, 1999), and the causes are pronounced in developing countries (Mukeka et al., 2019). In Africa, HWC increased due to climate change, habitat loss, and low PA management effectiveness (Tiller et al., 2021). HWC not only have adverse effects on communities’ livelihoods but also lead to negative attitudes toward wildlife conservation and wildlife resources (Nelson, Bidwell & Sillero-Zubiri, 2003). Managing competition between humans and wildlife for resources is a critical conservation issue (Woodroffe, Thirgood & Rabinowitz, 2005). The attitudes held by local people towards wildlife are critical to managing the conflicts (Naughton-Treves & Treves, 2005). To ensure the wildlife management policies are effective to the local condition, it is important to understand the attitude of the local people which provides an overview on HWC (Kideghesho, Røskaft & Kaltenborn, 2007). Assessment of the local people attitude can provides insight to respond to economic losses by wildlife and wildlife protection regulation, the degree to which they are willing to coexist with wildlife, and attitude surveys may also predict how people’s attitudes will influence conservation attitudes and vice versa (Tarrant, Kruger & du Preez, 2016; Browne-Nunez & Jonker, 2008).

The conflict between people and elephants is a special case of HWC (Draheim et al., 2015). Agricultural crop raiding is one form of human-elephant conflict (HEC) (Long et al., 2020; Sitati, Walpole & Leader-Williams, 2005; Sitati et al., 2003). Crop raiding has negative impacts on perceptions of local communities toward elephants, which can strongly undermine conservation efforts (Osborn & Hill, 2005). The abundance of African elephants is more linked with human livelihood activities (Hoare, 1999), and the human impact on elephant causes a great challenge to PA and wildlife managers, and the local people themselves (Sitati et al., 2003; Said et al., 2016). HEC is not a new phenomenon in African countries; however, due to encroachments of settlements onto elephant habitats, the conflict has continually increased (Hoare, 2007; Hoffmeier-Karimi & Schulte, 2015). The conflict between humans and elephants is a challenge in many African countries, leading to disturb the community livelihood (van Aarde & Jackson, 2007; Hoare, 1999) and brings a negative attitude toward their conservation efforts (Kamau, 2017; Osborn & Hill, 2005).

Human-elephant conflict (HEC) in Africa is more pronounced in village lands close to PAs within settlements and farmland-dominated areas (Mmbaga, Munishi & Treydte, 2017). Elephant crop raiding and encroachment of human activities into wildlife habitats as source of conflict were also increased over the past years in and around Kafta Sheraro National Park (KSNP) of the Tigray region (Kalayu et al., 2021; Samson, 2021; Atakilt, Gidey & Gebregizabher, 2016). However, up-to-date KSNP lacks details information about HEC and attitudes of the local communities toward elephant conservation which hinders effective conservation measures for the park and its existing wildlife. Therefore, the objectives of the present study include: (1) To investigate trends of KSNP natural resource utilization by communities (*i.e.,* as sources of HEC and a threat for elephant conservation), (2) assess the extent and trends of HEC, and methods used to minimize their negative impacts, (3) determine attitudes of the communities toward the conservation of the African elephant and KSNP, and (4) assess the effect of some demographic and socioeconomic factors on the attitude of local communities adjacent to KSNP.

## Materials & Methods

### Description of the study area

Kafta Sheraro National Park (KSNP) was designated as a park in 2007 (Letter, No: 13/37/82/611) with an area of 2,176.43 km^2^. The park was formerly named the “Shire Wildlife Reserve” and was established in 1973 with an estimated area of 750 km^2^. KSNP is located in the Kafta Humera and Tahitay Adiyabo districts of the Tigray region 1356 km from Addis Ababa and 490 km from Mekelle city. The park is situated in between latitude 14°05′–14°27′N and longitude 36°42′–37°39′E. The park is bordered by Eritrea in the north and traversed by the Tekeze River (Fig. S1). The elevation of the park varies from 539 to 1,130 m above sea level. The landforms are flat plain, undulating to rolling; some isolated hills and ridges, a chain of mountains and valleys. The climate is characterized by hot to warm semiarid and has seasonal rainfall (Fitsum & Bikila, 2020). The maximum monthly temperature is in April (43.7 °C), while the minimum monthly temperature is in December (19.2 °C) and January (19.1 °C). The mean monthly temperature ranges from 28.35 °C to 35.1 °C. The coolest temperature occurs in August, while the warmest temperature occurs from March to May. The rainfall pattern varies greatly with the months of the season. Short rains occur in June and September, and long rains occur during July (174 mm) and August (252 mm), whereas rare cases of rain in the remaining months appear (Fitsum & Bikila, 2020).

KSNP forest communities are broadly categorized as Acacia-Commiphora woodland and bushland proper with dominant *Acacia mellifera* and *Balanites aegyptiaca* species; Combretum-Terminalia woodland and wooded grassland with *Terminalia brownii* and *Boswellia papyrifera* as frequent species; and Riparian/riverine forest with *Hyphaene thebaica* as dominant species (Friis, Sebsebe & Breugel, 2010). The park harbored more than 70 woody species, 46 trees, 18 shrubs, and six tree/shrubs. The most dominant and frequent tree species of the park are *Acacia mellifera*, *Combretum hartmannianum*, *Terminalia brownii*, *Balanites aegyptiaca*, *Dicrostachys scinerea*, *Acacia senegal*, *Acacia oerfota*, *Boswellia papyrifera, Ziziphus spina-christi*, and *Anogeissus leiocarpus* (Fitsum & Bikila, 2020).

The park is home to 42 mammals, nine reptiles, 167 bird species, and fish and crocodile species. The presence of large mammals such as African elephant (*Loxodonta africana*), Caracal (*Felis caracal*), leopard (*Panthera pardus*), Greater kudu (*Tragelaphus strepsiceros*), oribi (*Ourebia ourebi*), waterbuck (*Kobus ellipsiprymnus*), spotted hyena (*Crocuta crocuta*), *Crocodile* sp., warthog (*Phacochoerus africanus*), aardvark (*Orycteropus afer*), Anubis baboon (*Papio anubis*), grivet monkey (*Chlorocebus aethiops*), and roan antelope (*Hippotragus equinus*) (Shoshani & Yirmed, 2008). In addition to the existence of wildlife, the hydrology of the Tekeze River makes KSNP a significant site for the priority of conservation (Fitsum & Bikila, 2020).

The livelihood and economic activities of the local communities depend on agriculture which is dominated by mixed farming of crop and livestock production. Sesame (*Sesamum indicum*), sorghum (*Sorghum bicolor*), finger millet (*Eleusine coracana*), teff (*Eragrostis tef*), maize (*Zea mays*), banana (*Muza species*), mango (*Mangifera indica*), papaya (*Carica papaya*), onion (*Allium cepa*), garlic (*Allium sativum*), potato (*Solanum tuberosum*), and chili pepper (*Capsicum annuum*) were the most important types of crops produced in the study area.

### Data collection

#### Field observation and questionnaire survey samples

This field assessment supplements the socioeconomic survey and was taken in different seasons and times to note HEC, elephant signs in the area, elephant groups involved in crop raiding, the time of raiding and left croplands (*i.e.,* during the day), control measures used by the local people and elephant responses. In the HEC survey, global positioning system (GPS) points were taken, which were hotspots for conflicts both in wet and dry seasons. Information on crop damage was also accessed to determine food preferences and the degree of crop loss. The field observations took place from January 2018 to September 2020 in both the wet and dry seasons.

A preliminary survey was conducted one week prior to actual data collection. This pretesting and piloting (before using it to collect data) observation helped to identify the park boundaries, decide the number of kebeles (Kebeles: the lowest governmental administrative units of Ethiopia) that they included, and understand the overall situation of KSNP. The survey was conducted with selected local household residents from two districts (Weredas); Kafta Humera and Tahitay Adiyabo. Seven kebeles were purposively selected and the selection of kebeles was based on the nearest to the park boundary, the experience of high resource utilization and encroachment by the community, and widespread observation of elephant crop raiding (crop damage). The Adiaser and Aditsetser kebeles are located within the eastern boundary of KSNP because these areas are more encroached by crop cultivation and animal grazing. The AdiGoshu, Mayweyni, and Wuhedet kebeles are located within the southern boundary of the KSNP, while Adebay and Freselam are adjacent (western part of the park) that possess elephant crop raiding is a negative important issue.

Households were systematically selected from each kebele. Thus, the first household selection started randomly from the settled households, and then the next households were selected systematically at every 9th interval in each kebele using the formula below until the given sample size was reached. Therefore, the size of the interval (k) for selection was calculated by k = N/n, where k = the size of the interval for selection; N = total population (households); and n = the number of samples required for the study (Fitsum, Bikila & Alemayehu, 2022). Finally, the sample of household heads was recorded as: AdiGoshu (*n* = 72), Mayweyni (*n* = 30), Wuhedet (*n* = 28), Adiaser (*n* = 37), Aditsetser (*n* = 70), Adebay (*n* = 124), and Freselam (*n* = 34), ranging from 6.5–21 km from the boundary of the park. For analysis, the selected kebeles were divided into two distance categories, as those less than 9.0 km from the boundary of the park were assigned as “close”, whereas those that were above 9.0 km were termed as “far away” (distant). The distances were determined by recording GPS points of the park boundary and kebeles and by calculating the distance between them through Arc Map 10.3 software. Even though, all seven kebeles surveyed were outside the park boundary, most of their farming practices were inside and in the buffer zone of KSNP.

#### Household socioeconomic and attitudinal response

The interview data were collected from November 2018 to June 2019 through face-to-face semi-structured questionnaires. Before the main data collection, the drafted questionnaire was amended prior to use and improved in quality (clarity) based on a pre-survey sample of 90 randomly selected individuals from seven kebeles that were finally excluded from the main sampled respondents. The purpose of the interview was to assess the interaction of the local communities with African elephant and KSNP. Open-ended questions were preferable to discuss and elaborate some issues raised about elephant conservation problems. Interviews were limited or selected to one family head, either male or female, of an individual household. The respondents who lived or existed before 1991 were considered native, while respondents who came from other areas of the region after 1991 were consider resettled (*i.e.,* relocated) because almost all those kebeles were well established after 1991.

Our questionnaire consisted of five basic questions/sections areas of interest. The 1st section covered the socio-demographic characteristics and economic activities of the respondents (*e.g.*, age, gender, level of education, household size, land holding size, land occupation type, alternative income) and park communities inter action (settlement condition and distance from settlement to park border). The 2nd investigated residents’ natural resource utilization from the park. The 3rd part asked the respondents to rank the problematic wild animal species (elephant), and rate the damage response (a = high problem, b = moderate problem, c = low problem, and d = no complaint/no problem), which helped us to know the effect of elephant damage and compare it with other wild animals. Moreover, participants who selected that wildlife/elephant posed a problem (*i.e.,* selected a, b, and c) had to identify a type of conflict in KSNP that the individual households experienced over the previous ten years. The 4th part interviewed about the situation of HEC: the elephant crop raiding trend as (a = increasing, b = same, c = decreasing, and d = unknown), the time of crops visited by elephant (a = Morning, b = night, c = afternoon, d = unknown), season (a = dry season, b = wet season, c=both seasons, d = no opinion), and the control mechanism or action taken (gun sound/banging noisy materials, lighting fire, alternative crop cultivation, and traditional fences) by the participants to prevent elephant crop damage. The 5th section explored their knowledge, attitude, and perception of the local community toward conservation of the KSNP and African elephants. To assess elephant and park conservation attitudes in the local communities, we employed 14 statements/questions about interviewees’ attitudes toward the conservation and benefits of the KSNP and the existing African elephant. The interviewees’ responses to each conservation attitude statement were categorized and recorded on a five basic point Likert scale (Likert, 1932) as: (strongly agree = 1, agree = 2, neutral = 3, disagree = 4, and strongly disagree = 5).

The demographic and socioeconomic information as well as park communities’ interaction at the individual household level were utilized as independent variables while dependent variables (*i.e.,* attitude toward elephant and park) in the logistic and generalized linear regression analyses employed in this paper. Unnecessary complication we avoided and questions were fitted with the respondents’ background and level of understanding. The questionnaire was conducted to sample households in two separate seasons of wet (June–November) and dry (December–May) of 2018–2019. The questionnaire was basically prepared in English languages (File S2); however, the interview was carried out in the Tigrigna language, which is spoken by all the people of the Tigray region.

#### Focus group discussion and key informant interviews

The household-based semi-structured questionnaire gathered data were supplemented and strengthened by interviewing focus group discussions (FGDs) on the seven selected kebeles, the Park management staff, and kebeles, wereda, and zone administrators, including professional experts. The total number of FGD respondents in the kebeles ranged from 12–38 individuals comprising AdiGoshu (26), Mayweyni (13), Wuhedet (12), Adiaser (15), Aditsetser (27), Adebay (38), and Freselam (14). The participatory FGD was emphasized on the awareness and attitude of the local residents to the conservation of KSNP and African elephants, HEC outside and inside the park.

Qualitative data were collected from a total of 32 individuals comprising two Western and North Western zone administrators, two wereda and seven kebele administrators, and 21 professional experts in crop production and natural resource management (*i.e.,* forestry and wildlife experts). The data collected from these key informants were assessed on elephant crop raiding impact in the local community and the practices for compensation feedback, the natural resources utilization approach, and the techniques to minimize the negative impacts for the sustainability of the park.

Moreover, the interview was conducted with a total of 27 park management staff consisting of 20 scouts, six field experts, and the park manager (one). These are front-line participants in the management and protection of African elephant in the area. The interview for park management staff was made to gather the information about the list of wildlife and estimate the number of elephants, the challenge faced to conserve the park in general and elephants in particular, their opinion to solve the encountered conservation problems, and the local attitude in relation to the conflict between humans and wild animals (elephants).

### Data analysis

#### Quantify household’s attitude

The attitudes toward elephant and elephant conservation in KSNP were estimated and coded in response to the attitude statement on a scale of strongly positive (1) to strongly negative (5) and the sum of the scales that gave a total attitude score (composite score) of each respondent. We generated a total of 14 attitude statement questions (*i.e.,* seven for elephant conservation and seven for park conservation attitudes measurement). The internal consistency (reliability) of the total scores was tested using Cronbach’s alpha (α) coefficients (α =between 0 and 1) and inter-item (between the seven statements) correlations (Cronbach, 1951). Finally, two separate indices (*i.e.,* attitude indices toward the conservation of elephant and KSNP) were calculated following (Cahyat, Gonner & Haug, 2007) manual guide for index calculation under IBM SPSS 26.0 (IBM Corp, Armonk, NY, USA) software. The “α” coefficients were evaluated using the criteria for acceptable range values > 0.7 (DeVellis, 2003) indicating good internal consistency. Statements (questions) that were internally inconsistent were removed following the guidelines outlined by George & Mallery (2003). The corrected inter-item (between the seven statements) correlation of individual statements > 0.5 was selected. The remaining statements of the composite score ensured and reflected the overall attitude toward elephant and park conservation. Our results indicated that “α” coefficient for the attitude index of both elephant and park was > 0.7.

### Statistical analysis

Descriptive statistics (means and % ages) were used to describe the demographic and socioeconomic features of the respondents. Nonparametric chi-square (*χ*^2^) and correlation were employed to test the statistical significance of the differences in the variables using the R-statistical Package (R-Development Core Team, 2019). Response variables were categorical either binomial (*e.g.*, conservation awareness Vs no awareness) or multinomial (agree, disagree, and neutral attitudes). A binary logistic regression model was employed to test the effect of socioeconomic factors (age, gender, education level, distance, and settlement condition) on respondents’ awareness/knowledge status toward park establishment and conservation objectives. Moreover, we developed generalized linear models (GLMs) regression to identify the best predictor variables or evaluate the impact of individual household age, gender, education, distance, land occupation type, and level of elephant crop damage (high, moderate, and low/no complaints) that influenced the respondents mean attitude score toward elephant conservation. Moreover, in the analysis of respondents’ attitude mean score responses to KSNP conservation, another set of independent variables was used that include age, gender, education level, distance, settlement condition, land holding size, land occupation type, and awareness status. All statistical analyses were performed using IBM SPSS statistics 26.0 (IBM Corp, Armonk, NY, USA) software with the level of significance set at *p* < .05.

### Ethics approval and research site permission

The study Ethics was reviewed and approved by College of Natural and Computational Science, Research Department Graduate Committee (DGC, 2019) and Ethiopian wildlife conservation authority Research Ethics Review Committee. Prior to field visit and data collection, the permission letter was gained from Ethiopian wildlife conservation authority for the selected study site of KSNP. Before distributed the questionnaire the objective of the study was briefly explained to participants. We surveyed based on the verbal consent of an individual and there was no experimental work on human subjects. The survey questionnaires were completed after getting a consent from the individual and thus as per the Addis Ababa University (AAU) system, as long as there is no human or animal experiment, we don’t need IRB approval. The questionnaire described below (File (S2)) excluded personal identifiers (privacy) like name of interviewee/respondents.

## Results

### Demographic characteristics and socioeconomic activities of respondents

The respondents varied in sex ratio, age category, level of education, and socioeconomic characteristics. Of the 395 respondents, 74.2% (*n* = 293) were male household heads and 25.8% (*n* = 102) were female participants. The respondent’s age categories covered from youngest (22 years) to oldest (75 years) with the average age of 44.9 years. There was a difference in the age categories of the respondents; more than 50.6% (*n* = 200) were between 22 and 39 years old, while 16% (*n* = 63) and 33.4% (*n* = 132) were between 40 and 57 years old and older than 58 years old, respectively. Many (59%) of respondents settled far away (*i.e.,* live greater than 9 km from the park boundary), whereas 41% (*n* = 162) settled close (*i.e.,* live within 9 km of the park boundary). The educational status of the respondents was different among respondents; 25.8% (*n* = 102) were informal education, 74.2% (293) had formal education (1–8th = 65.1% and 9–12th = 9.1% grades), and none of them were beyond secondary level education. 70.6% of the respondents had 4–6 household members. Moreover, 2–3 and 7–8 household members had 22.9% and 6.5%, respectively, with a mean of 4.5. The major livelihood and economic activities of the respondents were crop production (50%, *n* = 197), mixed crop and livestock (43.7%, *n* = 173), and livestock rearing alone (6.3%, *n* = 25).

### Local communities’ utilization of natural resources of KSNP

The local communities along the boundary of the KSNP utilized various natural resources from the park. A significant difference was observed among the respondents in terms of natural resource utilization in the park (*χ*^2^ = 74.33, *df* = 6, *p* < 0.001). The majority (74.58%) of respondents used the park as a means of grazing land, followed by 46.04% sources of fuel wood (Table S1). A total of 13.21% of the local community collected *Boswellia papyrifera* plants as sources of aromatic resin, which directly generates income. Woody plant species such as *Anogeissus leiocarpa*, *Dalbergia melanoxylon* and grass species are utilized as row material for house construction (30.38%) from KSNP. A total of 24.02% of the respondents gained different types of food sources, such as fish, wild honey, edible fruits of *Adansonia digitata*, *Tamarindus indica*, *Ziziphus spina-christi*, *Diospyros mespiliformis*, *and Ficus sycomorus*. Gold mining was another natural resource of the park used by the respondents. A total of 39.57% of the local people dominantly utilized the water source of the Tekeze River and its tributary rivers for the drinking of livestock and irrigation. The community also obtained other benefits, such as making of mats, bags, and baskets from *Hyphaene thebaica* tree plant, traditional medicinal plants (*e.g.*, *Plumbago zeylanica*), and sources of income (*e.g.*, selling fuel wood, fodder, and wild honey). According to the distance from the selected kebeles to the park, slight significant variation was observed among the seven kebeles in their natural resource consumption. For example, more than 93.0% of the Freselam and Wuhedet respondents obtained grazing for their livestock following fuel wood sources (90.86%) from Wuhedet, whereas 53.78% and 32.31% of the Adiaser respondents utilized grazing and fuel wood sources, respectively.

### Human-elephant conflict (crop raiding)

*Existing crop raiders and level of crop damage*: The total number of 395 household heads (∼10% from the total population of the seven kebeles) and focus group discussion interviews revealed that crop damage by wild animals has occurred continually for the past ten years; however, no respondents reported other HWC cases, such as injury to animals and humans or disease transmission to animals and humans. Qualitative and quantitative results indicated that crop raiding by elephant has continued to be a major problem in the study area over the last ten years (Fig. S2). The respondents announced that once elephants visit agricultural fields they severely damage the crops. Most respondents (*n* = 354, 89.62%) evaluated crop damage by elephant as “major”, whereas anubis baboon (*Papio Anubis*: 1.2%, *n* = 5) and porcupine (*Hystrix cristata*: 0.5%, *n* = 2) claimed as “moderate damage”, and none of the respondents evaluated as major and moderate damage by grivet monkey (*Chlorocebus aethiops*), common warthog (*Phacochoerus africanus*), and greater kudu (*Tragelaphus strepsiceros*). The status of these wild animal crop damages was categorized under minor and no problems because their negative impact on field crops of the local communities was practically negligible (Fig. S2). Thus, the respondents recognized that elephant is the most problematic crop pest wild animal in KSNP over the previous years. Therefore, the HWC discussed in our study focuses on elephant crop raiding and associated issues.

### Factors affecting elephant crop raiding

*Crop types and distribution* **:** Twelve species of crops were visited and damaged by elephant crop raiding incidence in KSNP. Among the most frequently raided cereal crops were maize (*Zea mays* L*.*), sorghum (*Sorghum bicolor* L.), teff (*Eragrostis tef*), finger millet (*Eleusine coracana* L.), and pearl millet (*Pennisetum typhoideum*). *Zea mays* and *Sorghum bicolor* were cross checked by the respondents the most preferable and vulnerable crop types by African elephant (*Loxodonta africana*). Moreover, vegetable types, such as potato (*Solanum tuberosum*), tomato (*Solanum lycopersicum*), okra (*Abelmoschus esculentus*), and duba (*Cucurbita pepo*), and fruits, such as papaya (*Carica papaya*), mango (*Mangifera indica*), and banana (*Musa species*), were among the crops attacked by elephant (Table S2).

*Season and time of crop raiding* **:** However, elephant crop raiding patterns occurred both in the dry and wet seasons; the majority of respondents believed that damage increased during the wet season. Of the total hectares of crop land damaged by African elephants during the surveyed period, most respondents (60.9%) answered that elephant visited their crops more frequently during the wet cropping season of rain fed crops, whereas 24.1% believed during the dry season of irrigated crops. Some households (15.6%), particularly those living in Adiaser and Aditsetser kebeles (*i.e.,* relatively farther kebeles from the park border), had no opinion regarding elephant season preferences, while none of the respondents chose both seasons for elephant crop raiding. The raids on rain fed cereal crops noticeably increased during the late wet season, whereas raids on vegetables and fruits increased during the long dry season. There was a significant difference between seasons in the size of damaged crop land (*χ*^2^ = 234.5, *df* = 4, *p* < 0.05). The crops visited by African elephant varied with time of daily pattern among kebeles (*χ*^2^ = 76.86, *df* = 3, *p* < 0.0001. The highest number of respondents (60.51%, *n* = 239) confirmed that elephant crop damage visited their field crops more frequently during the night (7:00 pm–6:00 am), and a few respondents (13.67%, *n* = 54) detected damage during the day both in the morning (6:00 am–10:00 am) and afternoon (3:00 pm–6:00 pm). Some of the respondents (25.82% *n* = 102) were unable to identify the exact time of crop raiding by elephant in their field crops (Fig. S3).

*Location of farmland and distance of residence to park*: The majority of respondents (72.19%, n=) revealed that the trend of elephant crop damage had ‘increased’, while 9.78%, n = respondents selected stable. Only 7.01%, n = respondents believed the damage was decreasing because those respondents trans-located their field from the park in the past ten-year period. Some respondents (11.01%) had no awareness or complaints about the change in crop damage because their farmland and settlement were far away from the border of the park). The trend of crop damage differed significantly (*χ*^2^ = 113.98, *df* = 3, *p* < 0.001) among the study kebeles (Table S3). Crop damage differed according to the location of household farmland and the distance from settlements to parks. The trend of crop damage was relatively high that a crop land found inside and at the periphery of the park compared to farmland located relatively far from the park boundary. The communities living far away from the park boundary are less influenced by elephant crop raiding than those living close to it. The number of respondents who complained about crop damage significantly affected (*p* < 0.001) the distance from settlements to parks. Around 55% of the respondents of the kebeles complained the most who settled less than 9.0 km (close) to the park. 45% of the respondents settled greater than 9.0 km (*i.e.,* relatively far) were stated to have low crop damage in their field crops (Table S3).

Regarding the extent of elephant crop damage over ten years, the respondents’ answers were categorized into four levels: high, moderate, low, and no problem/no complaint. The degree of crop damage and yield loss complaints varied significantly among the local communities (*χ*^2^ = 234.5, *df* = 3, *p* < 0.05). Most respondents (59.82%) reported that elephants were high crop damages, with an additional 16% reporting elephants as having no damage/no complaints. Of the seven kebeles, the highest complaint was perceived from Adebay (87.1%), followed by Freselam (70.6%), Wuhedet (67.86%) and Myweyni (60%), while respondents in Adigoshu, Adiaser, and Aditsetser had confirmed low and no complaints of the impacts of crop damage by African elephant. However, in Adebay, Freselam, Wuhedet, and Myweyni, which are the nearest kebeles to the border of the park, suggested that elephant poses the highest damage; while more than 30% of the respondents answered “no damage” by elephant in Adiaser and Aditsetser, which are relatively the farthest kebeles (Fig. S4).

### Local traditional techniques for preventing crop raiding

The local communities’ farmers adopted four major types of protecting approaches to minimize the amount of crop damage and yield loss by elephant crop raiding in KSNP. On average, there was a significant difference among the elephant crop raiding protection approaches (*χ*^2^ = 32.31, *df* = 3, *p* < 0.001). The major practices applied were pursuing the noise of gun and other local materials stimuli called ‘Wanchif’ (*i.e.,* Tigrigna name); physical barriers such as fencing of their crop land using locally available thorny Acacia and Ziziphus plant species, making trenches of wall; local repellents such as lighting of ‘Shig’ (*i.e.,* Tigrigna name) and flashlight around their farmland during the night and land use planning (*i.e.,* alternative crop farming systems), such as replacing the repeated cultivation of elephant-preferred crops (*e.g.*, *Zea mays* and *Sorghum bicolor*) with unpreferred crops (*e.g.*, *Sesamum indicum*). Most of the respondents (81.99%) agreed that noise of a gun and other related noise producing material (*e.g.*, drum, tin cans) mechanisms was the most common and effective protection method used to pursue the existing elephant crop raiding in all selected kebeles, followed by lighting fire and torch lights around the crop land (44.95%), alternative crop cultivation (37.21%), and physical barriers (30.80%). Some of the respondents (11.22%) did not express clear ideas (Table S4).

Respondents varied significantly (*χ*^2^ = 104.11, *df* = 4, *p* < 0.001) in their view of appropriate sustainable and coexistence management strategies in response to conflict with African elephant. Around 58% of the respondents suggested compensation from the government for the damaged crops; 24.1% wanted construction of barriers between the park boundary and farmland by the government. Others (10.0%) suggested the use of different traditional protection techniques to minimize the crop damage caused by African elephant, and 5.7% of respondents recommended resettlement/relocation of the farmland owner to other wildlife-free areas. Only 2.2% did not respond regarding the management techniques, and none of the respondents proposed killing of elephants as a solution to crop damage (Fig. S5).

### Locations of HEC areas (elephant crop raiding)

Human-elephant conflict (HEC) data were collected *via* repeated field surveys and interviews with respondents in the study area, *i.e.,* Kafta Sheraro National Park (KSNP). The field survey was conducted from 2018 to September 2020 to show the locations of HEC distributions and respondents who observed elephant crop damage from the past ten years of experience to recent years. According to the questionnaire survey of 395 interviewed households adjacent to the KSNP, on average, 72.2% of the respondents confirmed that HEC (*i.e.,* elephant crop raiding) increased in the area in the past ten years and that there was considerable crop damage (Table S3). More than 82% of the respondents mentioned that elephants damaged their seasonal and irrigated crops (Fig. S4). The conflict areas are located inside the KSNP and outside the park of adjacent agricultural fields. The respondents claimed that conflicts were common where the household’s agricultural fields (seasonal and riverside irrigated crops) were located inside and adjacent to the park boundary (Fig. S6).

### Resident’s knowledge/awareness about KSNP conservation

Most of the respondents (54.18%, *n* = 214) were aware of the aim of KSNP establishment and conservation of natural resources. However, the rest of the respondents (45.82%, *n* = 181) had little/no awareness regarding the conservation objectives of KSNP. The logistic regression model conducted on the response (dependent) variable of awareness/no awareness and five variables (*i.e.,* age, gender, education level, settlement condition, and distance between settlements and park) as independent variables. Gender (male, *p* < 0.001), education level (formal, *p* < 0.001), age category (*p* < 0.001), settlement condition (native, *p* =0.046), and distance from settlement to park (close to park, *p* = 0.037) (Table S5) The most significant independent variables, whereas household size, settlement duration and farm location excluded from the model equation were not determinant factors (*p* > 0.05) and did not influence the respondents’ awareness/knowledge difference on conservation of KSNP.

The model coefficients were significant (*χ*^2^=241.14, *df* = 6, *p* < 0.001) and the Nagelkerke R^2^ was 0.634. The prediction accuracy of the model was 81.5%. The significant variables in the model were explained as indicated in Table S5. The probability that males tended to respond to awareness was 3.535 times higher than that of female respondents. Males were more likely to have awareness about the aims of KSNP conservation and related issues. Furthermore, compared with the probability of interviewees aged >58 years responding “yes” (awareness), the probability of interviewees aged 22–39 and 40–57 years was 98.97 and 22.46 times, respectively. Respondents having primary and secondary education were 8.31 and 7.65 times more likely conservation awareness, respectively, than those respondents having informal education. Similarly, compared with the probability of resettlers choosing awareness, the probability of native respondents choosing awareness was 1.98 times higher. Respondents’ settlement close to the park (6.5 to 9 km) was 1.84 times conservation awareness than those who lived >9 km from the KSNP border.

### Community attitude on the conservation of KSNP and elephants

**Park**: The majority of the local communities supported the establishment and practice of KSNP conservation, respectively. This result indicated that the majority of the communities had positive attitudes toward the conservation of natural resources in the park. However, 60.5% of the interviewees agreed that the park had a positive impact on the conservation of natural resources, while around half of the interviewees did not perceive any benefits from the conservation of the park. Of the total respondents, 48.35% felt that KSNP conservation increased competition for resources among residents. More than half of the respondents complained that park conservation had led to conflict between the local people and the park managers (Table S6). The internal consistency of the seven questions was evaluated and measured using Cronbach’s alpha (α). The validity of the model indicated that all seven attitude statements concerning elephant conservation were included in the final results of the reliability analysis because all the questions belonged to the recommended correlation scale and had corrected interitem correlation scores (*i.e.,* how much each item correlated with the overall questionnaire score) greater than 0.5, while the final ‘α’ of the elephant attitude index was 0.90.

**Elephant**: Residents’ attitudes of elephant conservation were evaluated in this study through seven attitude statements. Our results showed that most of the interviewees (59.80%) felt that elephants were responsible for raiding cultivated crops inside and outside the park. Although more of the local people (44.81%) supported the existence of elephants in their communities, most of the respondents (46.43%) opposed increasing the number of elephants in KSNP. On the other hand, more than half of the respondents supported the existence of elephants in communities, which initiated tourist attractions and were generally important to park ecosystems. A total of 48.72% of the sampled households believed that elephants have a natural right to live in the area. The majority of the respondents (53.31%) also supported the developmental conservation practices proposed in the Construction of Water Reservoirs project in the elephant conservation habitat of the KSNP, which addresses water security, especially during the dry season (Table S6). The seven attitude statements toward KSNP conservation were addressed by individual households; however, our internal consistency evaluation model result showed that item statement three was excluded from the final result of the reliability of the attitude index because this question had corrected interitem total correlation scores less than 0.5. A decrease in this item increased the final ‘α’ of the KSNP attitude index. The index had six statements with a high ‘α’ of 0.89.

### Factors affecting attitudes toward KSNP and elephants conservation

**Park:** The variation in attitudes toward protected area (*i.e.,* KSNP) conservation was significantly influenced (*p* = 0.008) by respondents aged between 22 and 39 years (younger age) and 40 and 57 years (middle age), which positively impacted their attitudes (*p* = 0.029). The education level of the local people significantly (*p* < 0.001) explained the variation in this attitude, and those who attained formal education (*i.e.,* primary and secondary education) had a positive attitude. The distance from the settlement to the park significantly (*p* < 0.001) influenced the attitude of respondents toward park conservation, as the local communities that settled far (greater than 9 km) from the KSNP had more positive attitudes than those who settled close (6.5–9 km) to the park. Household experience knowledge was another significant determinant of residents’ attitudes toward the conservation of PA natural resources (*p* < 0.001) (Table S7).

**Elephant**: The variation in attitudes toward elephant and elephant conservation was significantly influenced (*p* = 0.007) by respondents aged between 22 and 39 years (younger age) and 40 and 47 years (middle age), which positively impacted their attitudes. The education level of the local people significantly (*p* = 0.003) explained the variation in this attitude, and those who attended formal education had relatively positive attitudes (Table S7). The distance from the settlement to the park significantly (*p* = 0.002) influenced the attitude of respondents toward elephant conservation, as the local community that settled far (greater than 9 km) from the KSNP had a more positive attitude than those who settled close (6.5 to 9 km) to the KSNP. Moreover, the two most important variables that significantly explained the variation in the interviewees’ attitude scores were the crop damage trend and the degree of crop loss. The respondents who reported an increase in elephant crop raiding in the past ten years had a significantly (*p* = 0.004) negative attitude toward elephant conservation, while those who reported a decrease and the same trend of crop raiding had a more positive attitude. The respondents who reported high and medium levels of crop damage had significantly negative perceptions (*p* < 0.001 and *p* = 0.013, respectively). Individual households that experienced low or no complaints about elephant crop raiding had more positive attitudes than those who experienced high or moderate crop damage (Table S7).

## Discussion

### Human-elephant conflict

HEC (*i.e.,* elephant crop raiding) has slightly increased over the past ten years in KSNP. HEC and elephant conservation threats (*e.g.*, traditional mining extraction, charcoal production, and grazing of livestock, *etc.*) were reported in KSNP (Kalayu et al., 2021; Samson, 2021; Atakilt, Gidey & Gebregizabher, 2016). The local community of KSNP gained fuel wood, grazing, income sources (gold and resin), and house construction materials. In Ethiopia PAs, natural resources utilization by the community was reported (Seyoum & Afework, 2021; Mekbeb, Kumara & Addisu, 2019; Aberham, Balakrishnan & Gurja, 2017b; Reddy & Sintayehu, 2014). The availability and scarcity of forest products in communities fringing PAs; does influence the attitudes of residents toward the protection of natural resources (Kideghesho, Røskaft & Kaltenborn, 2007). The expansion of agriculture and increased human and livestock numbers increased HWC in Ethiopia (Seyoum & Afework, 2021). Crop fields and settlements were vulnerable hotspot for HEC (Nad, Roy & Roy, 2022; Billah et al., 2021), and increasing agriculture in the boundary of a protected area (PA) posed a source of conflict (Bailey et al., 2016). Moreover, in Zimbabwe (Hoare, 1999), India (Bal et al., 2011), and Kenya (Sitati et al., 2003), HEC increased due to the expansion of farms.

Based on field observations of HEC and household interviews, maize (*Zea mays*) and sorghum (*Sorghum bicolor*) were reported as the most preferable and vulnerable crops by African elephant. The respondents’ suggestions and attentive field observations, those crops have a wide distribution and high nutritional acceptance by elephants. The vulnerability of crop types to elephant was reported in Africa (Mukeka et al., 2019; Kamau, 2017; Mmbaga, Munishi & Treydte, 2017; Mwakatobe et al., 2014; Hoare, 1999; Ogunjobi et al., 2018; Harich et al., 2013). Similar feeding preferences were also suggested for Asian elephant (*Elephas maximus*) (Webber et al., 2011; Neupane, Johnson & Risch, 2017).

### Determinant factors of elephant crop raiding

*Location of agricultural fields and the distance between settlements and* park: HWC is a pronounced issue in many African countries of local communities that live at the boundary of PAs (Angwenyi, Potgieter & Gambiza, 2021; Mukeka et al., 2019; Mmbaga, Munishi & Treydte, 2017). Crop raiding is the most prevalent form of HWC worldwide, and more than 70% crop damage has been recorded in Kenya (Long et al., 2020) and Zimbabwe (Lamarque et al., 2009). Households living close to KSNP were more influenced and complained by elephant-induced crop damage. In contrast, communities living relatively far away from the park were less affected by elephant crop raiding. Graham et al. (2009) and Mwakatobe et al. (2014) stated the proximity of settlements or crop fields to PAs increases elephant and other wildlife crop damage incidents. Similar results was reported in Ethiopia (Ayenew, Girma & Zerihun, 2019; Aberham, Balakrishnan & Gurja, 2017a; Yigrem, Wondimagegnehu & Hailu, 2016), Tanzania (Kiffner et al., 2021; Mwakatobe et al., 2014), and Kenya (Long et al., 2020). Crop damage by elephant also increased with shorter distance of farm to water point (Montero-Botey, Miguel & Perea, 2021). In our study, kebeles (villages) of Adebay, Freselam, Wuhedet, and Myweyni with above 60% levels of elephant crop damage were observed, because, elephants are visited more frequently in these areas and their farms are in proximity (6.5−9.5 km) to/from the park border. These kebeles are nearest to KSNP, and most of their agricultural crops are found inside and across the border of the Park. Crop raiding in the KSNP was higher where farmlands were found inside and nearest to the park (Mwakatobe et al., 2014; Reid, 2012; Graham et al., 2009; Blanc et al., 2007; Hoare, 1999). Similar findings reported in Africa including Ethiopia (Aberham, Balakrishnan & Gurja, 2017a; Holmern, Nyahongo & Røskaft, 2007; Patterson et al., 2004; Barnes et al., 2005; Monney, Dakwa & Wiafe, 2010) and Asia (Sampson et al., 2019).

*Seasonal and daily patterns of elephant crop raiding:* Elephant crop raiding follows both season and daily time patterns (Mmbaga, Munishi & Treydte, 2017). According to household responses and field observations, crop damage in KSNP was higher during the late wet season (*i.e.,* rainfall terminated) between September and November. Crop raiding cases peak after a wet season, as wild animals could be attracted to mature crops, (Mukeka et al., 2019; Mmbaga, Munishi & Treydte, 2017). The probable reason in our study was elephant crop raiding is related to phenology of crop plants (crops maturity and harvesting time) when they are most nutritious to them during the late wet season. As the wet season progresses and water is more readily available in all areas of the park, elephants are widely dispersed inside and outside the park. However, during the dry season, as water scarcity is very high, elephants are concentrated near the water points of KSNP (*i.e.,* Tekeze River), and only irrigated crops are available on the river sides that are easy raids by elephant and natural fodder availability and quality decline with field crops.

Elephant crop raiding in this study took place during the night (60.51%). Similar to our study, 95% of elephant crop damage occurred during the night and early morning (Mmbaga, Munishi & Treydte, 2017) and crop raiding incidents occurred during the hours of darkness (Sitati, Walpole & Leader-Williams, 2005).

### Local prevention and control measures of elephant crop raiding

The local communities of KSNP applied several traditional controlling methods (gun sound and beating drums and cans, lighting fire and flashlight, alternative crop cultivation, and construction of physical barriers) to prevent elephant damage to their crops. Mmbaga, Munishi & Treydte (2017) indicated that shouting, banging on iron sheets, tins, and drums whistles and lighting fires, torch light) were used as elephant repellents. Night burning fires and banging tin and drums combined with guarding effort prevent elephant crop raiding (Sitati, Walpole & Leader-Williams, 2005). The most preferable prevention measures for elephant-induced damage were physical barriers (Sampson et al., 2019), patrolling (Su et al., 2020) and growing alternative crops (Neupane, Johnson & Risch, 2017). However, guarding was the best controlling mechanism to prevent crop damage from larger wildlife (*i.e.,* elephant) (Aberham, Balakrishnan & Gurja, 2017a). Cultivation of less preferred crops by elephant, such as pepper (*Capsicum annuum*), was common to minimize elephant crop raiding (Kamau, 2017; Pozo et al., 2017; Monney, Dakwa & Wiafe, 2010; Webber et al., 2011; Parker & Osborn, 2006). In KSNP elephants favored certain crops above others and avoided certain crops (*e.g.*, sesame and cotton) (Table S2). Combination of strategies may be the most effective way to minimize crop damage by elephants (Sitati & Walpole, 2006; Hoare, 2012).

The degree of effectiveness of elephant crop damage control methods suggested that guarding together with noise, lighting fire and flashlight methods are effective in curbing elephant-induced damage. However, physical barriers such as locally available materials to fence their crops land were less effective as elephants were easily able to break through the fences. Furthermore, in our study, this method was difficult to apply for producers who had large farm sizes. A study reported from east Africa that local barriers alone are more effective for smaller crop raiders (Sitati, Walpole & Leader-Williams, 2005) if farmland totally enclosed by electrified fences (O’Connell-Rodwell et al., 2000).

### Knowledge/awareness of the local community toward KSNP

The awareness of the local communities was affected by age categories, gender, education level, settlement condition, and distance between settlements and parks. Male respondents had higher park conservation awareness than females; this might be because females have less contact and low participation in nature conservation and related community issues. The communities also considered women to always be fit to household work and family care. This made them lack the confidence to participate equally with males in all community activities. Similar findings reported that females were less likely to express their perceptions of problems and associated benefits in four PAs of Myanmar (Allendorf & Allendorf, 2013). Another study in Tara National Park, Serbia, stated that females had poor relationships and less awareness about the park (Tomicevic, Shannon & Milovanovic, 2010). In contrast to our study, gender differences did not vary respondents’ awareness of the conservation of protected areas in Tanzania (Hariohay et al., 2018). However, the respondents in the age categories of 22 to 39 years and 40 to 57 years had more awareness about parks and the aim of park conservation. This group has enough knowledge about the aim of KSNP establishment, and most of these categories have achieved formal education than the other age groups. The low level of awareness indicated by the age group above 58 years might be due to a lower/informal education level, as more than thirty percent of the respondents had not achieved formal education. Similarly, older respondents had low awareness and poor relationships with PA (Tomicevic, Shannon & Milovanovic, 2010). Other findings in Chitwan National Park, Nepal, agreed that age differences affected respondents’ awareness and attitudes (Carter et al., 2013). Moreover, a similar trend in Rungwa Game reserve, Tanzania (Hariohay et al., 2018) and Serengeti National Park (Lyamuya et al., 2016) indicated that the local people’s knowledge of conservation varied with their age categories.

The education level of the respondents also influenced their awareness of park conservation, and those who had formal education were more aware than those who had informal education. The respondents with formal education took natural resources courses and probably created a variation in awareness due to their capacity to read different types of nature-related conservation books and periodic newsletters. The positive impacts of education on PAs were reported by (Hariohay et al., 2018; Tomicevic, Shannon & Milovanovic, 2010). The settlement of households in proximity to the KSNP border had more awareness than those far away settlements because this might be posed through continuous contact with the park. Local community residences close to Serengeti National Park were more aware of wildlife conservation (Bitanyi et al., 2012). Community residents close to the four nature reserves in South Africa had more interaction with these reserves than those far away from the areas (Angwenyi, Potgieter & Gambiza, 2021). The native (non-resettler) respondents were more aware of PA conservation because households who had lived there and their livelihood depended on natural resources for a long period of time had more experiences than those short lived (resettlers) to the area. According to Hariohay et al. (2018), local people living close to PAs and native to the area boundary were more knowledgeable than living far from PAs and resettlers, respectively (note: resettler: voluntary relocation of peoples from pre-existing area to new area in order to improve and secure their livelihood sustainability).

### Local community’s attitude toward KSNP and elephant conservation

The local communities adjacent to KSNP had mixed perceptions (positive and negative attitudes) toward the park and elephant conservation. In our study, the communities have predominantly a positive attitude toward park and wildlife conservation despite the restriction of access to resource use they do not get any economic support from the park, the level of crop damage by wildlife/elephants, and conflicts with park management, because, these issues were believed and brought a negative perception of the local communities toward conservation. The communities agreed with most of the conservation statements that measured their perceptions and attitudes. Our results concur with a study in African PAs (Ardiantiono et al., 2021; Abukari & Mwalyosi, 2018; Gandiwa et al., 2014). Despite local tensions, communities positively supported wildlife conservation in Ethiopia PAs (Seyoum & Afework, 2021; Ayenew, Girma & Zerihun, 2019; Aberham, Balakrishnan & Gurja, 2017a; Mekbeb et al., 2010; Yigrem, Wondimagegnehu & Hailu, 2016). In other African countries, the local communities were more likely to have a positive attitude toward PAs and wildlife conservation. In South Africa, communities positively supported the sustainable conservation of reserves; however, they had conflicts with reserve staff due to restrictions on resource use, which negatively impacted their livelihood (Angwenyi, Potgieter & Gambiza, 2021). However, as Guerbois et al. (2013) reported, immigrants closely connected with park resources expressed a more negative attitude toward PA. As Abukari & Mwalyosi (2018), Synman (2014) and Allendorf (2007) reported that local residents in communities near PAs in developing countries do appreciate the non-economic value of PAs (*i.e.,* ecological and socioeconomic values). People residing adjacent to the PA had a positive attitude toward elephants, as they received economic benefits from ecotourism and improved mitigation practices (Neupane, Johnson & Risch, 2017). Positive attitude might be achieved through environmental education from PA authorities, ecotourism and willingness to learn about non-harmful wildlife control (Seoraj-Pillai, 2016; Lindsey et al., 2005).

### Factors influencing community attitudes toward KSNP and elephant conservation

Gender, age categories, education level, distance between settlements and parks, and awareness status significantly influenced the communities’ attitude response toward conservation of KSNP and elephants. However, landholding size, settlement condition, and land occupation type, did not play a significant role in predicting community residents’ attitude difference. The age categories from 22 to 39 years and 40 to 57 years were more likely to have a positive attitude toward KSNP and elephants than those in advanced age groups (over 57 years) because the third age (>57 years) category is a references category. The decrease in positive attitude with an increase in age of respondents might be because aged people had their own crop land inside the park, including livestock, and most of them did not attain formal education. Furthermore, aged residents around KSNP have low awareness toward significant of PAs and its conservation. The findings concur with Hariohay et al. (2018)*,* who reported that households with aged (*i.e.,* more than 55 years) were mostly owners of livestock and that crop farms had a negative perception of wildlife conservation. However, a significant positive correlation between age and PA and wildlife/elephant conservation was observed (Ochieng, Elizabeth & Nigel, 2021). Aged respondents had a little awareness toward elephant and its conservation (Ardiantiono et al., 2021). However, negative correlation between attitude and age of respondents was also reported against our results (Guerbois et al., 2013; Khatun, Ahsan & Røskaf, 2012).

Attitudes and perceptions of gender significantly influenced the conservation of PAs and their corresponding resources (Ochieng, Elizabeth & Nigel, 2021; Deng et al., 2015). From the present study communities’ point of view, males were dominantly participating in agriculture and were more exposed to outdoor field work, while females kept at home work and cared for the whole family and children. As a result, male household heads had a more positive attitude toward PA and wildlife conservation than females, and this positively correlated with their level of knowledge/awareness about PA (Ardiantiono et al., 2021; Mir et al., 2015; Allendorf & Allendorf, 2013). Education positively influenced the attitude of the communities toward the conservation of PAs and wildlife. These findings are in conformity with studies by other scholars (Rahman, Abdullah Al Mahmud & Shahidullah, 2017; Parker et al., 2014; Carter et al., 2013; Barthwal & Mathur, 2012), who suggested that formal/more educated respondents were more likely to support the establishment and conservation of PAs and wildlife conservation. Sarker & Røskaft (2014) reported that most respondents with illiterate/informal education did not support the conservation of PAs and elephants. Local residents with formal education are more likely to obey rules protecting PAs than illiterates (Bragagnolo et al., 2016). Educated residents have the opportunity to employ, which provides alternative income that reduces the dependency of their livelihood on PAs natural resources (Kideghesho, Røskaft & Kaltenborn, 2007).

The distance of settlement to the PA boundary determines the level of interaction between humans and elephant/KSNP conservation. The results of our study indicated that the distance between residence and KSNP had a significant effect on the conservation attitude of respondents. Local communities that lived far away from the park showed a more positive attitude than those that lived close to KSNP because they experienced less interaction with wildlife and rarely visited elephant in their field crops. In this case, most respondents living in the administrative units of Adiaser and Aditsetser kebeles are relatively far away from the park boundary, showing a positive conservation attitude. On the other hand, the majority of residents who lived in Adebay, Freselam, and Wuhedet kebeles close to the park border showed a negative attitude. This directly reflects residents experiencing frequent crop damage by elephants, a lack of alternative income, and restricted access to the park resources made the community to bring negative attitude toward conservation. Similar results were reported from African PAs (Ochieng, Elizabeth & Nigel, 2021; Guerbois et al., 2013; Abukari & Mwalyosi, 2018; Sarker & Røskaft, 2014), where respondents’ settlement far away from the PA positively influenced conservation attitudes, whereas settlements near to PAs negatively influenced the conservation attitude as the HWC is high. However, the far-distance residents of this study rarely reported conflicts with elephants, and their interaction was limited to specific areas and seasonal elephant’s observations. This statement is consistent with Guerbois et al. (2013), who found that residents who had frequent contact with elephants had more negative attitudes than those who had rare contact. Likewise, communities close to PAs that have negative conservation attitudes were due to the damage induced by problematic animals (Kioko et al., 2008), and they did not gain any visible benefits from the conservation practices (Bennett & Dearden, 2014). The main drivers of negative attitudes toward conservation were household location (*i.e.,* living close to the PA boundary), losses due to wild animals, and restricted access to natural resources (Guerbois et al., 2013). However, Deng et al. (2015) and reported that in contrast to our results, they found that residents’ proximity to the reserve border positively affects their attitude toward the conservation of the reserve because the local residents benefited from development projects that might result in more job opportunities and have less dependence on the resources of the reserve. Rahman, Abdullah Al Mahmud & Shahidullah (2017) outlined, residents who lived away from PA were less aware of the importance of conservation than those who lived close to the park did not favor the conservation practices of the PA.

Respondents’ knowledge about establishments and aims of PAs conservation was a significant factor in determining their attitudes toward the KSNP conservation. Respondents who knew and appreciate the objectives of KNSP conservation were likely to have a more positive attitude than their counterparts who have little awareness. This is inlined with Abukari & Mwalyosi (2018) and Allendorf (2007) who reported that the residents who know the objectives of PAs conservation to be considerate are more likely to hold positive attitudes toward PAs.

According to the resident’s location to elephant conservation area and elephant crop raiding experience; they reported different conservation attitude scores. The current study indicated that respondents who damaged their crop fields by elephant use expressed a more negative attitude toward elephant conservation. Similarly, scholars confirmed that residents in areas where there was negative interaction between humans and elephants (*e.g.*, elephant crop raiding) were likely to lead to less favorable (*i.e.,* more negative) attitudes toward elephant and its conservation (Ardiantiono et al., 2021; Neupane, Johnson & Risch, 2017). Elephant crop raiding has increased tension between Park and local communities, especially residents who have experienced crop loss by elephant grazing (Andyono et al., 2018; Oelrichs, Lloyd & Christidis, 2016). Crop damage or livestock depredation incidents had a significant negative influence on the attitude of local communities toward wildlife conservation (Hariohay et al., 2018; Mir et al., 2015). The negative attitudes of communities were brought as a result of economic damage by elephant crop raiding (Bandara & Tisdell, 2002). Residents who lived with wildlife had a negative attitude about wildlife, as HWC has been a serious threat (Long et al., 2020). The existence of crop damage and little elephant revenue provided to locals resulted in a negative perception of the communities toward the conservation of elephants (Taruvinga & Mushunje, 2014). Conversely, the positive interaction between elephant and human that received benefits from ecotourism and conservation activities might improve the local communities’ positive attitude (Sarker & Røskaft, 2014). Local people who lived adjacent (0–5 km) to PAs had a positive attitude toward the conservation of PA, to get some kind of benefit and to expect to share revenue from the reserve in the future, regardless of the existing cases of crop raiding (Mojo et al., 2018).

Farming type (*i.e.,* subsistence and commercial agriculture) is another important factor to determine the respondent’s attitudes toward wildlife and PA conservation. Subsistence and commercial agricultural farmers held both positive and negative attitudes toward wildlife and they affect by HWC in different ways (Seoraj-Pillai, 2016). In sub-Saharan Africa, studies show that negative attitudes toward wildlife exist among commercial (Parker et al., 2014; Lindsey et al., 2005) and subsistence (Gusset et al., 2008) farmers, especially toward carnivores (Parker et al., 2014; Gusset et al., 2008). In addition, a large group of elephants causes significant damage both to subsistence and commercial farms (Sitati & Walpole, 2006). In this area, elephants negatively impact subsistence farmers by damaging the crops they rely upon for their livelihoods and food security (Wittemyer, 2001) and their economic stability disturb (Sitati, Walpole & Leader-Williams, 2005). However, commercial farmers may be able to discourage depredation of crops using fencing and lethal control, such resources are unaffordable to subsistence farmers. Dickman (2010) showed subsistence farmers were antagonistic toward wildlife since the consequences of crop depredation following intensified by the lack of alternate income. According to Seoraj-Pillai (2016), household income negatively influenced attitudes this arises because subsistence farmers were lower income earners than commercial farmers. In contrast, some subsistence farmers indicated a positive attitude to wildlife for example; traditional land use ethics and the values of the residents who co-existed with wildlife for many centuries could play a role in shaping a positive attitude toward subsistence farmers (DeGeorges & Reilly, 2008). Commercial farmers had a positive attitude toward wildlife because crop damage by elephant decreased in areas with private investors (Montero-Botey, Miguel & Perea, 2021).

## Conclusion & Recommendation

Human**-**elephant conflict (HEC) caused by crop damage has increased in and around KSNP over the past decade, presenting a conservation challenge. The observed increase in elephant crop attacks was associated with LULC changes mainly caused by the expansion of settlements and the conversion of woodlands to croplands. Crop damage peaked during the late wet season when crops were ready for harvesting. The crop damage increased as the area of residential farmland closed the PA boundary. The residents of the KSNP had both negative and positive attitudes toward the park and elephant conservation. A negative attitude was associated with restrictive access to the natural resources of the park, crop damage by elephants, and an absence of benefits from conservation activities. Education level, distance between residences and park, and degree of elephant crop damage were major factors that significantly affected residents’ attitudes toward the conservation of KSNP and elephants. Elephant conservation attitudes significantly varied with the distance between residences and park, where local communities living very close to PAs and with frequent elephant crop damage occurrences had more negative attitudes toward elephant conservation. On the other hand, residents who lived relatively far from the PA border had more positive conservation attitudes than those living near the PA border. Therefore, the conservation objective of PA/wildlife might be achieved by rational discussion with the local communities rather than by forced means. Consequently, it is imperative to understand the attitude of local people and increase their level of awareness/knowledge of PA and wildlife conservation in particular and nature conservation in general through continuous education. The smooth coexistence of KSNP and wildlife with the local communities could be maintained by establishing buffer zones in the area to ensure conservation sustainability and community livelihoods. Financial support should be allocated to the local communities to use green energy such as electricity, solar energy, and biogas to minimize household dependence on forest resources. Together with the conservation practices encourage ecotourism and nature reserve activities so that the local communities are exposed to alternative job opportunities and profit sharing. Facilitating the compensation process for crop damage caused by elephant use may improve attitudes.

##  Supplemental Information

10.7717/peerj.19428/supp-1 Supplemental Information 1 Raw data

10.7717/peerj.19428/supp-2 Supplemental Information 2 Supplemental Tables

10.7717/peerj.19428/supp-3 Supplemental Information 3 Supporting file 1 figures

10.7717/peerj.19428/supp-4 Supplemental Information 4 Detailed socioeconomic study questionnaire form for households in English languages

## Additional Information and Declarations

### Data Availability

The following information was supplied regarding data availability: The raw data and questionnaire are available in the Supplementary Files.
