# Supplementary material for: Human-elephant conflicts and attitude of the local communities toward African elephant (Loxodonta africana) conservation in Kafta Sheraro National Park, Tigray region, Ethiopia
Source: PeerJ. 2025 May 22;13:e19428. doi: 10.7717/peerj.19428 (PMC12103844; doi:10.7717/peerj.19428)
Supplement: Supplemental Information 2 [file peerj-13-19428-s002.zip › Table1.docx]

**Table 1** Types of natural resources proportion by percentage used by the local communities in relation to distance from selected settlement to park who are found adjacent to Kafta Sheraro National Park (N=395) Notes: the parentheses indicate the average grazing time per kebeles

| Kebele | Distance (km) | Natural resources types used (%) | | | | | | | |
| --- | --- | --- | --- | --- | --- | --- | --- | --- | --- |
|  |  | Gold mining | Resin  collection | Fuel wood | Grazing  livestock | Food  sources | Water sources | House  CM | Others |
| Adebay | 7.0-9.5 | 21.31 | 8.34 | 50.23 | 78.57(4.5m) | 24.34 | 42.62 | 32.34 | 9.68 |
| Adiaser | 17.5-20 | 14.45 | 17.56 | 32.31 | 53.78(4.6m) | 21.20 | 39.81 | 29.34 | 5.40 |
| Adigoshu | 13.0-15.5 | 19.81 | 20.41 | 37.34 | 68.59(5.0m) | 24.32 | 43.00 | 31.23 | 11.11 |
| Aditsetser | 18.0-21 | 17.82 | 9.50 | 27.09 | 59.58(4.2m) | 19.54 | 38.33 | 27.50 | 5.71 |
| Freselam | 7.0-9 | 18.23 | 11.30 | 90.86 | 93.45(5.5m) | 26.12 | 38.51 | 33.52 | 8.82 |
| Myweyni | 8.0-10 | 16.16 | 11.73 | 41.05 | 72.00(5.0m) | 25.21 | 37.06 | 28.60 | 10.00 |
| Wuhedet | 6.5-8.5 | 18.02 | 13.62 | 43.43 | 95.60(5.4m) | 27.45 | 37.72 | 30.12 | 10.70 |
| **Average** |  | 18.00 | 13.21 | 46.04 | 74.51 | 24.02 | 39.57 | 30.38 | 8.77 |

CM = construction materials, m=months,

Note: However, the total number of sample of respondents was 395; over counts are predictable due to multiple responses of households to questions.
