# Supplementary material for: Human-elephant conflicts and attitude of the local communities toward African elephant (Loxodonta africana) conservation in Kafta Sheraro National Park, Tigray region, Ethiopia
Source: PeerJ. 2025 May 22;13:e19428. doi: 10.7717/peerj.19428 (PMC12103844; doi:10.7717/peerj.19428)
Supplement: Supplemental Information 2 [file peerj-13-19428-s002.zip › Table2.docx]

**Table 2** Percentage proportion of households in the seven kebeles who indicated elephant-induced crop damage on different types of crop species during the dry and wet seasons in KSNP between 2018 and 2019 (N=177)

| Crop name | Kebeles | | | | | | |
| --- | --- | --- | --- | --- | --- | --- | --- |
|  | Adebay | Adiaser | Adigoshu | Aditsetser | Freselam | Myweyni | Wuhedet |
| *Zea mays* | 86.36 | 42.10 | 38.29 | 23.33 | 34.48 | 16.67 | 26.31 |
| *Sorghum bicolor* | 89.09 | 47.37 | 55.32 | 43.33 | 82.76 | 66.66 | 68.42 |
| *Eragrostis tef* | 0.00 | 21.05 | 8.51 | 33.33 | 10.34 | 8.69 | 0.00 |
| *Eleusine coracana* | 1.82 | 31.58 | 12.76 | 30.00 | 13.79 | 13.04 | 5.26 |
| *Pennisetum typhoideum* | 2.73 | 0.00 | 0.00 | 0.00 | 3.45 | 4.35 | 10.52 |
| *Solanum tuberosum* | 6.36 | 10.52 | 4.25 | 0.00 | 0.00 | 0.00 | 0.00 |
| *Solanum lycopersicum* | 4.54 | 0.00 | 6.38 | 6.67 | 0.00 | 0.00 | 0.00 |
| *Abelmoschus esculentus* | 9.09 | 5.26 | 0.00 | 0.00 | 3.45 | 4.35 | 5.26 |
| *Cucurbita pepo* | 11.82 | 0.00 | 2.13 | 3.33 | 0.00 | 4.35 | 5.26 |
| *Mangifera indica* | 0.00 | 15.79 | 10.64 | 0.00 | 6.89 | 0.00 | 0.00 |
| *Carica papaya* | 22.73 | 0.00 | 17.02 | 0.00 | 0.00 | 0.00 | 0.00 |
| *Musa species* | 0.00 | 10.52 | 14.89 | 10.00 | 0.00 | 0.00 | 0.00 |

Note: Percentages in the table include only repeated crop raided households
