# Supplementary material for: Human-elephant conflicts and attitude of the local communities toward African elephant (Loxodonta africana) conservation in Kafta Sheraro National Park, Tigray region, Ethiopia
Source: PeerJ. 2025 May 22;13:e19428. doi: 10.7717/peerj.19428 (PMC12103844; doi:10.7717/peerj.19428)
Supplement: Supplemental Information 2 [file peerj-13-19428-s002.zip › Table3.docx]

**Table 3** The proportion of the households’ response toward the trend of crop damage by the existing African elephant during the past ten years inside and near Kafta Sheraro National Park

| Kebele | Distance (km) | Sampled household (N) | Trend of crop damage by African elephant (%) | | | |
| --- | --- | --- | --- | --- | --- | --- |
|  |  |  | Increased | Stayed the same | Decreased | Unknown |
| Adebay | 7.0-9.5 | 124 | 87.09 | 3.24 | 2.42 | 7.25 |
| Adiaser | 17.5-20 | 37 | 62.16 | 13.51 | 10.82 | 13.51 |
| Adigoshu | 13.0-15.5 | 72 | 66.67 | 12.50 | 9.72 | 11.11 |
| Aditsetser | 18.0-21 | 70 | 61.43 | 12.86 | 10.00 | 15.71 |
| Freselam | 7.0-9.0 | 34 | 82.35 | 5.89 | 2.94 | 8.82 |
| Myweyni | 8.0-10 | 30 | 70.00 | 13.33 | 6.67 | 10.00 |
| Wuhedet | 6.5-8.5 | 28 | 78.57 | 7.15 | 3.57 | 10.71 |
| **Average** | --- | --- | 72.19 | 9.78 | 7.01 | 11.01 |
