# Supplementary material for: Human-elephant conflicts and attitude of the local communities toward African elephant (Loxodonta africana) conservation in Kafta Sheraro National Park, Tigray region, Ethiopia
Source: PeerJ. 2025 May 22;13:e19428. doi: 10.7717/peerj.19428 (PMC12103844; doi:10.7717/peerj.19428)
Supplement: Supplemental Information 2 [file peerj-13-19428-s002.zip › Table4.docx]

**Table 4** Proportion of the selected respondents who described the protection mechanism on elephant crop raiding in and around Kafta Sheraro National Park during 2018-2019 of the study period (N=395)

| Kebele | N | Methods of protection (%) | | | | |
| --- | --- | --- | --- | --- | --- | --- |
|  |  | Gun sound & banging noisy materials | Lighting  fire & flashlight | Land use planning (alternative crop cultivation) | Local barriers  (traditional fences) | No opinion  about the methods |
| Adebay | 124 | 92.74 | 56.45 | 44.35 | 37.09 | 0.00 |
| Adiaser | 37 | 78.37 | 40.54 | 32.43 | 24.32 | 21.62 |
| Adigoshu | 72 | 77.78 | 41.67 | 38.90 | 30.55 | 8.52 |
| Aditsetser | 70 | 74.28 | 38.57 | 28.57 | 22.86 | 24.28 |
| Freselam | 34 | 85.29 | 50.00 | 40.54 | 35.29 | 9.02 |
| Myweyni | 30 | 83.33 | 43.33 | 40.00 | 33.33 | 7.85 |
| Wuhedet | 28 | 82.14 | 44.12 | 35.71 | 32.14 | 7.26 |
| **Average** | --- | 81.99 | 44.95 | 37.21 | 30.80 | 11.22 |

Note: N=Sampled households

However, the total number of sample of respondents was 395; over counts are predictable due to multiple responses of households to questions.
