# Supplementary material for: Human-elephant conflicts and attitude of the local communities toward African elephant (Loxodonta africana) conservation in Kafta Sheraro National Park, Tigray region, Ethiopia
Source: PeerJ. 2025 May 22;13:e19428. doi: 10.7717/peerj.19428 (PMC12103844; doi:10.7717/peerj.19428)
Supplement: Supplemental Information 2 [file peerj-13-19428-s002.zip › Table5.docx]

**Table 5** Binary logistic regression model analysis with awareness/no awareness of the conservation of KSNP as the response variable and age, gender, education level, settlement condition, and distance between settlements and parks as explanatory variables (N=395)

| Independent variables | *B* | S.E | Wald | df | *Sig.* | Exp (B) (95%C.I) |
| --- | --- | --- | --- | --- | --- | --- |
| Gender |  |  |  |  |  |  |
| Male | 1.263 | .323 | 15.26 | 1 | .000 | 3.535 (1.87-6.66) |
| Female* | - | - | - | - | - | - - |
| Age (years) |  |  | 79.15 | 2 | .000 |  |
| 22**-**39 | 4.595 | .522 | 77.50 | 1 | .000 | 98.97 (35.6-275.3) |
| 40**-**57 | 3.112 | .492 | 40.08 | 1 | .000 | 22.46 (8.57-58.86) |
| > 58* | - | - | - | - | - | - - |
| Education level |  |  | 42.54 | 2 | .000 |  |
| Primary (1**-**8^th^) | 2.118 | .331 | 40.89 | 1 | .000 | 8.31 (4.34-15.91) |
| Secondary (9**-**12^th^) | 2.035 | .572 | 12.68 | 1 | .001 | 7.65 (2.50-23.47) |
| Informal* | - | - | - | - | - | - - |
| Settlement condition |  |  |  |  |  |  |
| Native | 0.68 | 0.35 | 4.96 | 1 | .046 | 1.98 (1.0-3.45) |
| Resettler* | - | - | - | - | - | - - |
| Distance b/n settlement & park |  |  |  |  |  |  |
| 6.5**-**9.0 km | 0.610 | .293 | 4.332 | 1 | .037 | 1.84 (1.04-3.27) |
| > 9.0 km* | - | - | - | - | - | - - |
| Constant | -5.624 | .645 | 76.02 | 1 | .000 | 0.004 |

Note: * Set as reference variables (variables that have considered little influence on the variation in household awareness of KSNP natural resource conservation)**;** Significance test at (*p*<0.05, *p*<0.01, *p*<0.001)**;** Entry and removal of variables was screened by the Wald forward stepwise method with probability of 0.05 and 0.1, and finally, three variables were eliminated from the equation**;** A positive coefficient (*B*) variable indicates a higher likelihood of conservation awareness.
