# Supplementary material for: Human-elephant conflicts and attitude of the local communities toward African elephant (Loxodonta africana) conservation in Kafta Sheraro National Park, Tigray region, Ethiopia
Source: PeerJ. 2025 May 22;13:e19428. doi: 10.7717/peerj.19428 (PMC12103844; doi:10.7717/peerj.19428)
Supplement: Supplemental Information 2 [file peerj-13-19428-s002.zip › Table6.docx]

Table 6. Households’ response to the attitude statement toward the conservation of KSNP and African bush elephants

| I. **Attitude statements toward Kafta Sheraro National Park** | Agree | Neutral | Disagree |
| --- | --- | --- | --- |
| 1. From the beginning i supported the establishment of KSNP | 49.87 | 12.15 | 37.97 |
| 2. I support the practices of KSNP conservation | 52.91 | 14.18 | 32.91 |
| 3. KSNP has a positive impacts on natural resources conservation | 60.50 | 10.63 | 28.87 |
| 4. KSNP conservation have brought positive change on the local  community livelihood | 27.35 | 23.04 | 49.61 |
| 5. Conservation of the whole district area brings happiness | 60.00 | 16.71 | 23.29 |
| 6. KSNP conservation stabilizes communities utilization of NR | 38.74 | 12.91 | 48.35 |
| 7. The relationship b/n community and park managers is good | 34.17 | 11.65 | 54.18 |
| II. **Attitude statement toward African bush elephant** | Agree | Neutral | Disagree |
| 8. I support the existence of elephants in our community | 44.81 | 16.46 | 38.74 |
| 9. I encourage to increase the no of elephants in the area | 42.85 | 10.71 | 46.43 |
| 10. Conservation of elephants can open a door for tourist  attraction | 56.77 | 27.37 | 15.85 |
| 11. Elephants crop raiding doesn’t a significant issue in the area | 17.04 | 23.16 | 59.80 |
| 12. Elephants have the right to live in the area | 48.72 | 14.10 | 37.18 |
| 13. Elephants are important to the whole KSNP ecosystem | 51.91 | 19.59 | 28.50 |
| 14. Construction of water reservoirs assure elephant conservation | 53.31 | 10.20 | 36.48 |

(NR=Natural resources)
