# Supplementary material for: Human-elephant conflicts and attitude of the local communities toward African elephant (Loxodonta africana) conservation in Kafta Sheraro National Park, Tigray region, Ethiopia
Source: PeerJ. 2025 May 22;13:e19428. doi: 10.7717/peerj.19428 (PMC12103844; doi:10.7717/peerj.19428)
Supplement: Supplemental Information 2 [file peerj-13-19428-s002.zip › Table7.docx]

Table 7. Factors influencing local people’s attitudes toward KSNP and elephant conservation

| Kafta Sheraro National Park conservation | | | | | African bush elephant conservation | | | |
| --- | --- | --- | --- | --- | --- | --- | --- | --- |
| Independent variables | *B* | *S.E* | Wald χ^2^ | *P value* | *B* | *S.E* | Wald χ^2^ | *P value* |
| Age categories (years) |  |  |  |  |  |  |  |  |
| 22**-**39 | **+**0.47 | .18 | 7.1 | .01 | **+**0.026 | .190 | 0.02 | .007 |
| 40**-**57 | **+**0.24 | .16 | 2.3 | .03 | **+**0.12 | .17 | 0.59 | .74 |
| > 58 * | - | - | - | - | - | - | - | - |
| Education level (grade) |  |  |  |  |  |  |  |  |
| Primary (1**-**8^th^) | **+**0.76 | .13 | 34.3 | .00 | **+**0.08 | .132 | 0.41 | .003 |
| Secondary (9**-**12^th^) | **+**1.37 | .21 | 40.7 | .00 | **+**0.4 | .22 | 3.36 | .021 |
| Informal* | - | - | - | - | - | - | - | - |
| Distance b/n  settlement & park (km) |  |  |  |  |  |  |  |  |
| 6.5**-**9.0 | -0.43 | .11 | 16.0 | .00 | -0.238 | .12 | 3.99 | .002 |
| > 9.0* | - | - | - | - | - | - | - | - |
| Settlement condition |  |  |  |  |  |  |  |  |
| Long term lived | **+**0.06 | .12 | 0.3 | .60 |  |  |  |  |
| Short term lived * | - | - | - | - |  |  |  |  |
| Landholding size (ha) |  |  |  |  |  |  |  |  |
| 1-3.5 ha | **+**0.02 | .11 | 0.02 | .88 |  |  |  |  |
| > 3.5 ha* | - | - | - | - |  |  |  |  |
| Awareness condition |  |  |  |  |  |  |  |  |
| Aware | **+**0.7 | .15 | 21.9 | .00 |  |  |  |  |
| Not aware* | - | - | - | - |  |  |  |  |
| Crop damage trends |  |  |  |  |  |  |  |  |
| Increase |  |  |  |  | -0.75 | .27 | 7.65 | .004 |
| Stayed same |  |  |  |  | **+**0.02 | .23 | 0.01 | .87 |
| Decrease/no opinion* |  |  |  |  | - | - | - | - |
| Crop damage level |  |  |  |  | - | - | - | - |
| High |  |  |  |  | -1.1 | .25 | 20.3 | .00 |
| Medium |  |  |  |  | -0.6 | .25 | 5.55 | .01 |
| Low/no complain* |  |  |  |  | - | - | - | - |
| Intercept | 3.07 | .17 | 307 | .00 | 1.7 | .26 | 40.5 | .00 |

Note:^*^references categories (>58 years old, female, informal education, resettled, >9km distance, land size >3.5 ha, landowner, no awareness, decrease/no opinion, and low/no complain on crop damage)

A positive (**+**) value for “*B*” indicates a positive impact of the variable on the attitude of the respondent, whereas a negative (-) value indicates a negative impact. χ^2^=Chi-square
