# Supplementary material for: Human-elephant conflicts and attitude of the local communities toward African elephant (Loxodonta africana) conservation in Kafta Sheraro National Park, Tigray region, Ethiopia
Source: PeerJ. 2025 May 22;13:e19428. doi: 10.7717/peerj.19428 (PMC12103844; doi:10.7717/peerj.19428)
Supplement: Supplemental Information 3 [file peerj-13-19428-s003.zip › SuppFigures/Figure 1.pdf]

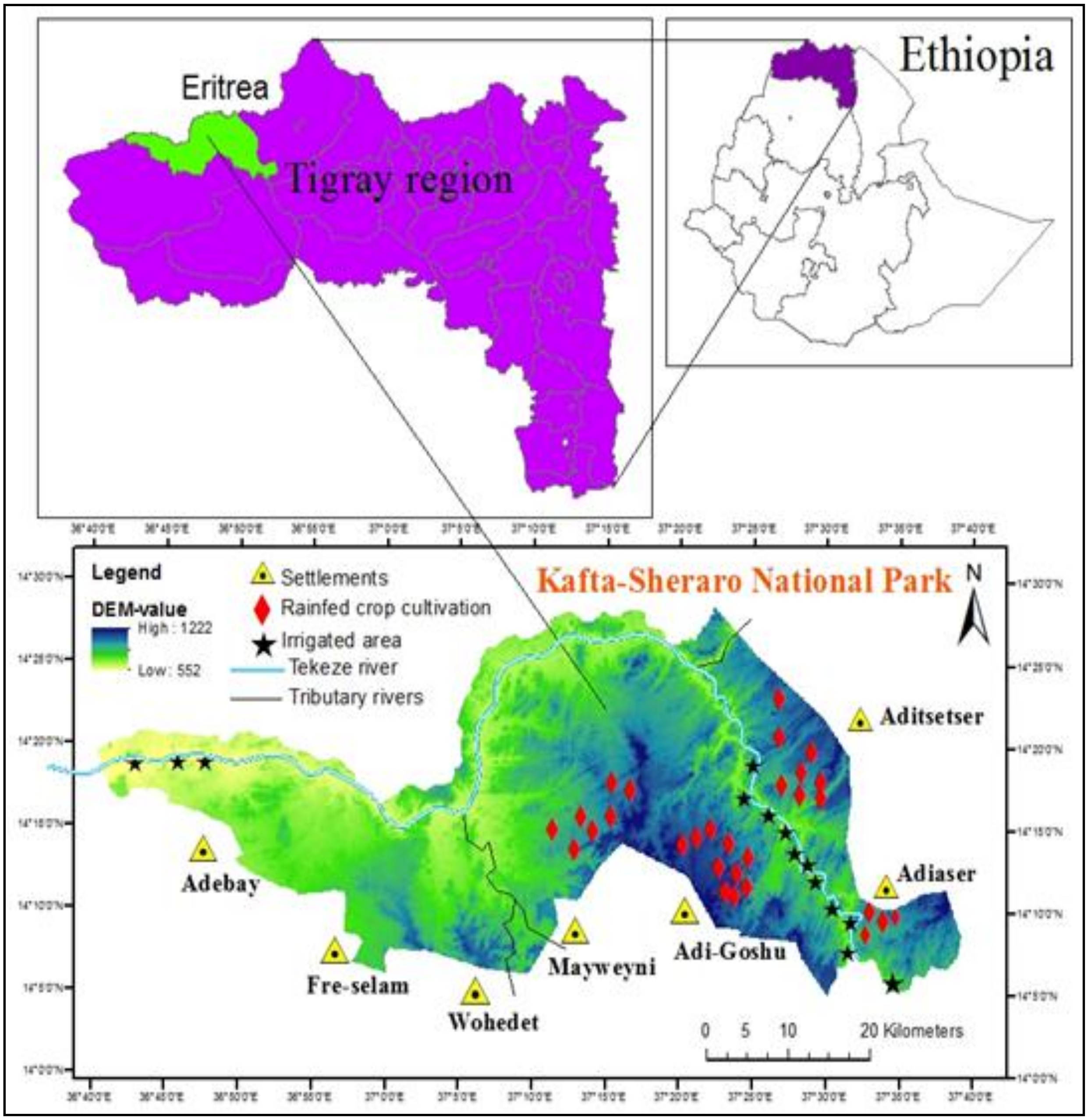

Figure 1. Location and elevation map of Kafta Sheraro National Park (KSNP) in the Tigray region (Fitsum et al., 2022). **Note**: The red/green color is created simply to differentiate the items one from the others and increase visibility, however, doesn't change any conceptual meaning on the entire map.
