# Supplementary material for: Human-elephant conflicts and attitude of the local communities toward African elephant (Loxodonta africana) conservation in Kafta Sheraro National Park, Tigray region, Ethiopia
Source: PeerJ. 2025 May 22;13:e19428. doi: 10.7717/peerj.19428 (PMC12103844; doi:10.7717/peerj.19428)
Supplement: Supplemental Information 3 [file peerj-13-19428-s003.zip › SuppFigures/Figure 2.pdf]

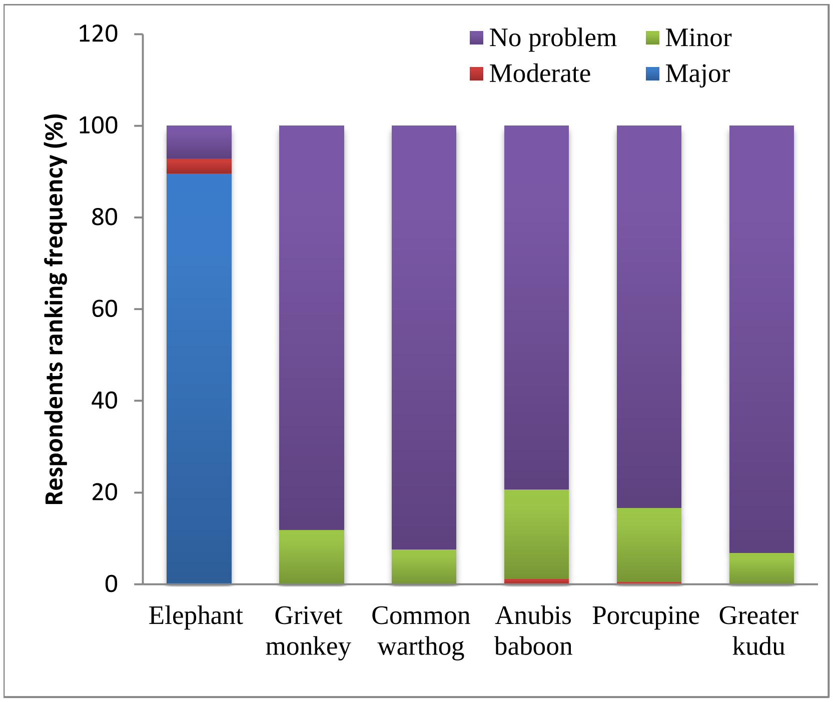

Figure 2. Local community recognized the existing crop raiders and the level of damage caused by each wild animal species. Ranking by (N=395) respondents from seven villages around KSNP between the 2018 and 2019 surveys. **Note:** The red/green color is created simply to differentiate the items one from the others and increase visibility, however, doesn't change any conceptual meaning on the entire map.
