# Supplementary material for: Human-elephant conflicts and attitude of the local communities toward African elephant (Loxodonta africana) conservation in Kafta Sheraro National Park, Tigray region, Ethiopia
Source: PeerJ. 2025 May 22;13:e19428. doi: 10.7717/peerj.19428 (PMC12103844; doi:10.7717/peerj.19428)
Supplement: Supplemental Information 3 [file peerj-13-19428-s003.zip › SuppFigures/Figure 3.pdf]

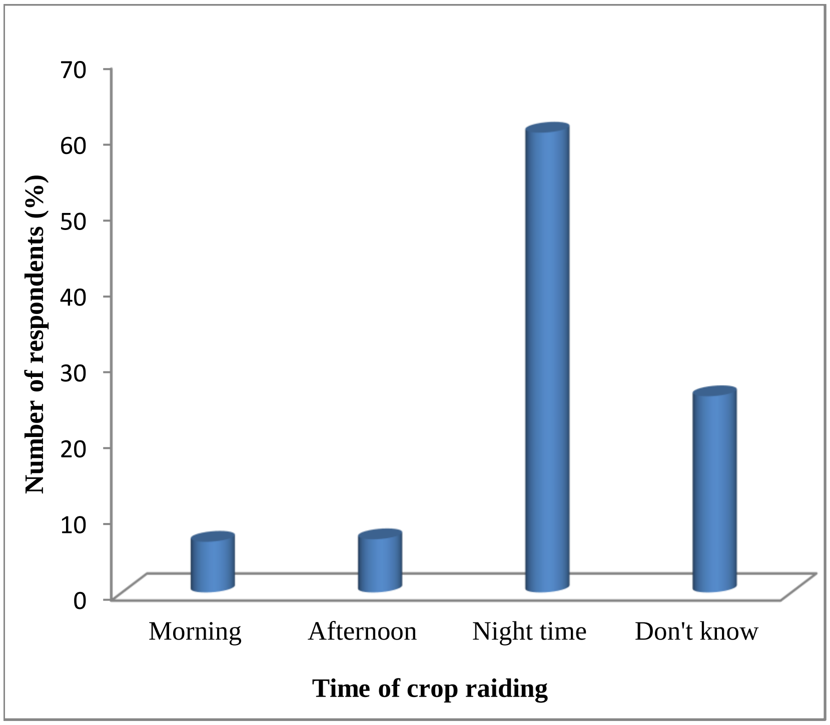

Figure 3. The local community field crops visited by African elephant inside and at the periphery of Kafta Sheraro National Park. Note: morning: 6:00 am-10:00 am; afternoon: 3:00 pm-6:00 pm; & night: 7:00 pm-5:00 am.
