# Supplementary material for: Human-elephant conflicts and attitude of the local communities toward African elephant (Loxodonta africana) conservation in Kafta Sheraro National Park, Tigray region, Ethiopia
Source: PeerJ. 2025 May 22;13:e19428. doi: 10.7717/peerj.19428 (PMC12103844; doi:10.7717/peerj.19428)
Supplement: Supplemental Information 3 [file peerj-13-19428-s003.zip › SuppFigures/Figure 4.pdf]

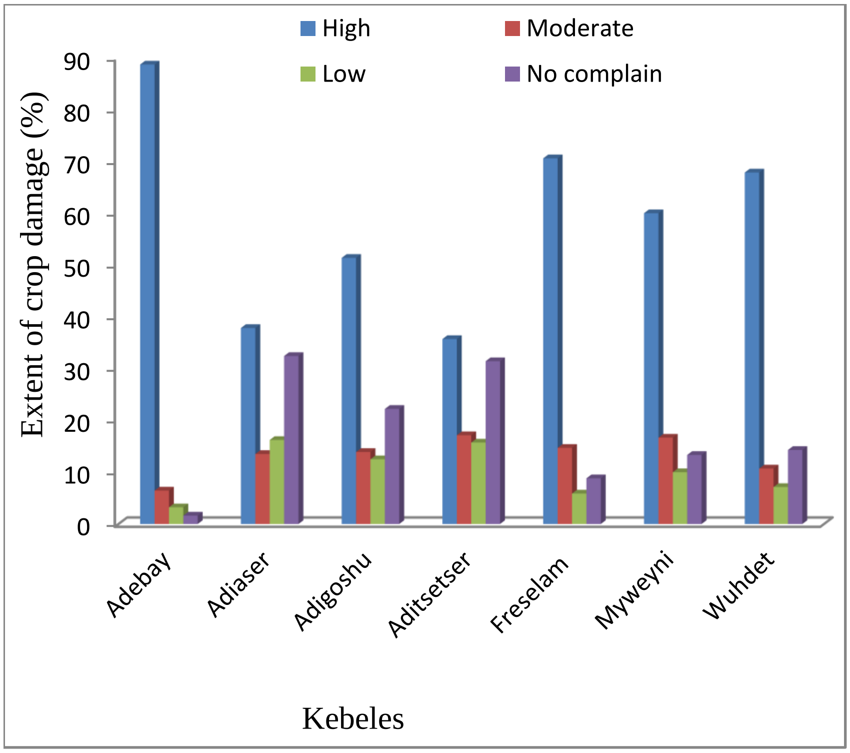

Figure 4 Percentage of the respondents who described African elephant crop raiding & their extent (level) of the damaging impacts of field crops (high=significant damage, moderate = medium damage, low= insignificant damage, & no complaint=absence of crop damage) **Note:** The red/green color is created simply to differentiate the items one from the others and increase visibility, however, doesn't change any conceptual meaning on the entire map
