# Supplementary figures and images for: Human-elephant conflicts and attitude of the local communities toward African elephant (Loxodonta africana) conservation in Kafta Sheraro National Park, Tigray region, Ethiopia
Source: PeerJ. 2025 May 22;13:e19428. doi: 10.7717/peerj.19428 (PMC12103844; doi:10.7717/peerj.19428)

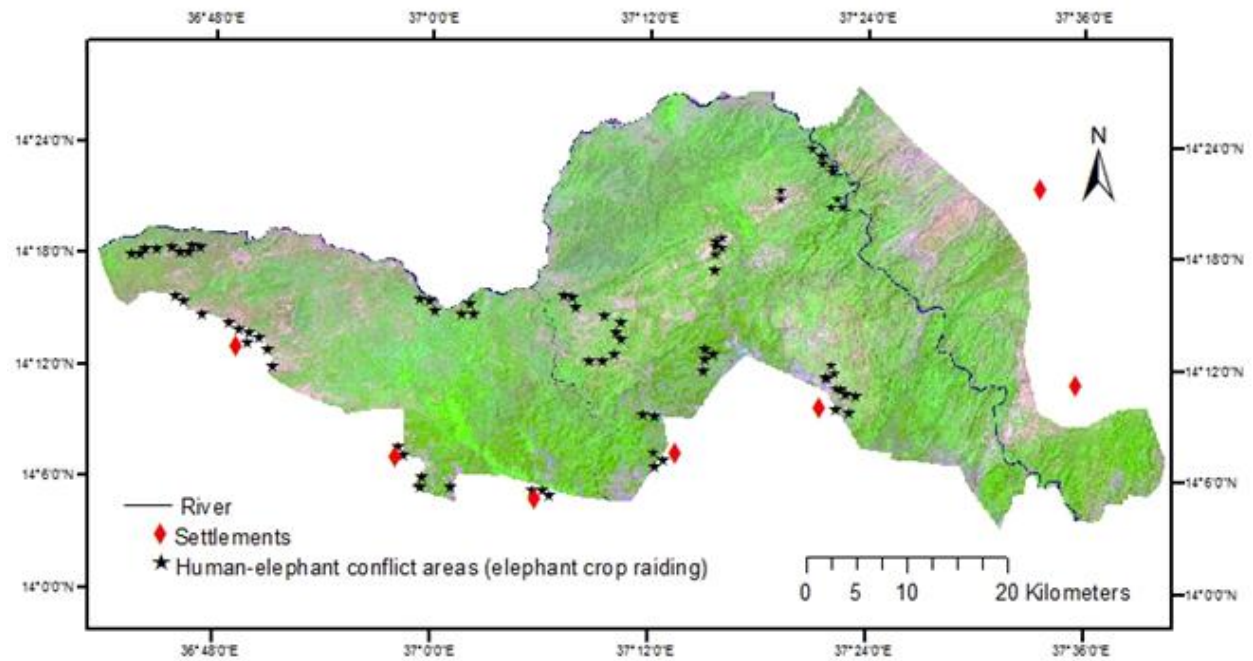

Figure 6. Locations of human-elephant conflict/elephant crop raiding areas in KSNP

Supplement: Supplemental Information 3 [file peerj-13-19428-s003.zip › SuppFigures/Figure6.pdf]
