# Supplementary material for: Human-elephant conflicts and attitude of the local communities toward African elephant (Loxodonta africana) conservation in Kafta Sheraro National Park, Tigray region, Ethiopia
Source: PeerJ. 2025 May 22;13:e19428. doi: 10.7717/peerj.19428 (PMC12103844; doi:10.7717/peerj.19428)
Supplement: Supplemental Information 4 [file peerj-13-19428-s004.docx]

**Table S1**

**Questionnaire for households (in English language)**

**Community-based open and closed questions for individual household heads in the Kafta Sheraro National Park (KSNP) Tigray region (November 2018-June 2019)**

Serial no of the interviewee: _________Date (date/month/year): ___________

GPS: X ___________Y____________Time taken________

The major aim of this research questionnaire is to understand how the local residents living surrounding Kafta-Sheraro National Park (KSNP) interact with park wildlife and its whole natural resources, which is directly interlinked with their livelihood. The specific purpose is to collect relevant data regarding the background information of the respondents, the attitude and perception of the local communities toward park and wildlife (elephant) conservation, human-elephant conflicts, land use/land cover history, and drivers of land use/land cover change in KSNP. This questionnaire is part of a PhD thesis that is being conducted by Fitsum Temesgen (a PhD student from Addis Ababa University, Ethiopia). Therefore, you are kindly requested to provide correct information for questions that you want to answer. Tick ('X') symbol if it is needed for each question response.

**1. Background information (demographic & economic activities) of the interviewee**

1.1 Gender (Tick ('X'))**:**

| Male |  | Female |  |
| --- | --- | --- | --- |

1.2 Age**:**

| < 20 | 21-35 years | 36-50 years | 51-65 years | above 66 years |
| --- | --- | --- | --- | --- |
|  |  |  |  |  |

1.3 Administrative description**:**

| Kebele | Wereda | Zone | Region | Bihereseb(Ethnicity) |
| --- | --- | --- | --- | --- |
|  |  |  |  |  |

1.4 Marital status (Tick ('X'))**:**

| Single | Married | Divorce |
| --- | --- | --- |
|  |  |  |

1.5 Household members/family sizes**:**

1.6 Occupation**:**

1.7 Educational status (level)**:**

| Informal | Formal | | | | | |
| --- | --- | --- | --- | --- | --- | --- |
|  | Grade 1-4 | Grade 5-8 | Grade 9-12 | Diploma | Degree | Others…….. |
|  |  |  |  |  |  |  |

1.8 For how long have you been living in this area?

<10 yrs 11-20 yrs 21-30 yrs 31-40 yrs above 41 yrs

1.9 Have you lived in other area previously?

Resettler =0 Native=1

1.10 If your answer is “yes=1” when was the resettlement period?

| Before 1991 | 1991-2007 | (2008-20018/19) Recently |
| --- | --- | --- |
|  |  |  |

1.11 What is your main source of livelihood and economic activities? (Tick ('X'))

| Sources of livelihood and income | Response |
| --- | --- |
| 1. Irrigated crops production |  |
| 2. Rain fed crop cultivation |  |
| 3. Livestock rearing |  |
| 4. Free natural resources collection |  |
| 5. Others (specify)………………… |  |

1.12 Do you have any other alternative sources of income?

No=0 Yes=1

1.13 If your answer is ‘yes=1’, list the types of work activities you occupied.

1.14 What are your main sources of energy for household consumption (specifically for cooking purposes?)

| Electricity | Fuel wood | Dung | Gas | Others…… |
| --- | --- | --- | --- | --- |
|  |  |  |  |  |

1.15 How far do you live/your settlement from the border of park area?

| <5 km | 5-10 km | 10-15 km | 15-20 km | >20 km |
| --- | --- | --- | --- | --- |
|  |  |  |  |  |

1.16 Where is your Farmland placed?

| 1. Inside the park | 2. Outside (near) the park (meter/km) |
| --- | --- |
|  |  |

1.17. Land type:

| 1. Owner | 2. Land less (Rent from someone) |
| --- | --- |
|  |  |

1.17 What is the type and size of your farmland occupied?

| Type of farmland used | Response | Size (hectare) | Land use permit card |
| --- | --- | --- | --- |
| 1. Irrigated land |  |  |  |
| 2. Rain fed crop cultivation L. |  |  |  |

1.18 What are the types of agricultural crops that are growing in your farmland?

Give a specific common name of the plants.

| Crops category | List of common and scientific names |
| --- | --- |
| 1. Cereals crops |  |
| 2. Fruits |  |
| 3. Vegetables |  |
| 4. Oil and industrial crops |  |

1.19 For what purpose and use have you grown the crops?

| Crop use | Names of the crop types |
| --- | --- |
| 1. Food source (home consumption) |  |
| 2. Cash crop (sale) |  |
| 3. Both for food and cash |  |

1.20 Do you want to leave the area or stay here?

Stay Leave

1.21. If you are happy to leave the area what are the challenges will expect? (Tick ('X'))

| Challenges | Response (Rank) |
| --- | --- |
| 1. Lacks alternative cultivated land |  |
| 2. Lacks choice of grazing land |  |
| 3. Lacks water for irrigation |  |
| 4. Disturbs livelihood style |  |
| 5. Destructs huge fruit plantation |  |

1.22 Which natural resources do you require access from the protected area? **(**Tick ('X')**)**

| Resources accessed | Response |
| --- | --- |
| 1. Grazing |  |
| 2. Firewood and charcoal production |  |
| 5. Gold mining |  |
| 6. House construction materials and related |  |
| 7. Gum and resin collection |  |
| 8. Food collection (Wild honey, edible fruits, fish) |  |
| 9. Water sources |  |
| 10. Medicinal plants collection |  |
| 11. Others….. |  |
|  |  |

1.23 If your answer is grazing for how long your cattle graze in the park? **(**Tick ('X')**)**

| **<** 3 months | 3-5 months | 5-8 months | The whole year (12 months) |
| --- | --- | --- | --- |
|  |  |  |  |

1.24 Is there any chance that the Eritrean community entered the park? **(**Tick ('X')**)**

No=0 Yes=1

1.25 If your answer is ‘yes=1’ in which season does the Eritrean community cross the park border?

| Season | Response |
| --- | --- |
| 1.Dry season |  |
| 2.Wet season |  |
| 3.Both |  |

1.26 If your answer is ‘dry season=1’, what is the reason for crossing?

1.27 How would you evaluate the relationship between your community and management of Kafta Sheraro National Park? **(**Tick ('X')**)**

| Excellent | Good | Satisfactory | Poor | Bad | Don’t know |
| --- | --- | --- | --- | --- | --- |
|  |  |  |  |  |  |

1.28 What are the problems faced with having cropland inside and near the KSNP? **(**Tick ('X')**)**

| Problems | Response (Rank) |
| --- | --- |
| 1. Damage crops by wildlife |  |
| 2. Loss of land utilization and livelihood |  |
| 3. Limited on access to resource use in the park |  |
| 4. Conflict with park staff |  |
| 5. Others (specify) |  |

**2. Awareness and attitudes of the local communities toward park habitat and wildlife conservation, particularly African elephant**

2.1. Do you have any knowledge/awareness about protected areas ̸ National Parks?

| No=0 |  | Yes=1 |  |
| --- | --- | --- | --- |

2.2 Would you support the establishment of Kafta Sheraro National Park?

| No=0 |  | Yes=1 |  |
| --- | --- | --- | --- |
|  |  |  |  |

2.3 If you support what are the benefits of Kafta Sheraro National Park?

2.4. If you didn’t support what is your reason for Kafta Sheraro National Park?

2.5 Do you think the establishment of Kafta Sheraro National Park has a positive impact on the conservation of natural resources?

| No=0 |  | Yes=1 |  |
| --- | --- | --- | --- |

2.6 Have you ever seen elephants in and outside Kafta Sheraro National Park?

| No=0 |  | Yes=1 |  |
| --- | --- | --- | --- |

2.7 Do you think the presence of elephants in this area has any benefit for the people?

| No=0 |  | Yes=1 |  |
| --- | --- | --- | --- |

2.8 If your answer is “yes=1”, what are the benefits of elephants? List them.

2.9 How many elephants would you estimate living in Kafta Sheraro National Park?

| Less than 50 | 50-100 | 100-150 | 150-300 |
| --- | --- | --- | --- |
|  |  |  |  |

2.10 Have there been noticeable changes in elephant and other wild animal numbers over the

past ten years?

| Increased/increase (**+**) | Decreased/decrease (-) | Stayed/stay the same | Do n’t know |
| --- | --- | --- | --- |
|  |  |  |  |

2.11 If there has been an increase in numbers, what do you think are the causes?

| Causes | Response (Rank) |
| --- | --- |
| 1. Decreases disturbance |  |
| 2. Seasonal change |  |
| 3. Diet availability |  |
| 4. Water availability |  |
| 5. Increases conservation activity |  |

2.12 Are elephants seasonally moving to other places or border countries?

No=0 Yes=1

2.13 If elephants are trans-boundary, in which season do elephants move?

| Season | Response | Season | Response |
| --- | --- | --- | --- |
| 1. Early dry season |  | 3. Early wet season |  |
| 2. Late dry season |  | 4. Late wet season |  |

2.14 If your answer is ‘late dry season’, what is the main root cause for movement of elephants to border country during this period?

| Causes | Rank |
| --- | --- |
| 1.Dry season fire hazard |  |
| 2.Scarcity of food |  |
| 3.Riverside vegetation disturbance by cultivation |  |
| 4.Drought |  |
| 5.Others (specify) |  |

2.15 For how many months more elephants stayed/stay in the park?

1-5 months 6-10 months 10-12 months

2.16 Do you think elephants and other wild animals should be protected in this area?

No=0 Yes= 1 explain why?

**3. Summary of attitude and perception of the sampled households on conservation of African elephant and Kafta Sheraro National Park (KSNP)**

According to the following conservation initiatives, how is your attitude and feeling of choice based on the degree statement of the agreement/disagreement? Choose and put your feedback according to the five Likert-type scale response categories as (1=strongly agree, 2=agree, 3=no opinion (neutral), 4=disagree, & 5=strongly disagree).

| **Attitude statement toward KSNP conservation** | | | Likert scale | | | | |
| --- | --- | --- | --- | --- | --- | --- | --- |
|  |  |  | 1 | 2 | 3 | 3 | 5 |
| 3.1 | From the beginning i supported the establishment of KSNP | |  |  |  |  |  |
| 3.2 | I support the practices of KSNP conservation | |  |  |  |  |  |
| 3.3 | KSNP has a positive impacts on natural resources conservation | |  |  |  |  |  |
| 3.4 | KSNP conservation have brought positive change on the local community livelihood | |  |  |  |  |  |
| 3.5 | Conservation of the whole district area brings happiness | |  |  |  |  |  |
| 3.6 | KSNP conservation stabilizes communities utilization of NR | |  |  |  |  |  |
| 3.7 | The relationship b/n community and park managers is good | |  |  |  |  |  |
| **Attitude statement toward elephant conservation** | | | 1 | 2 | 3 | 4 | 5 |
| 3.8 | | I support the existence of elephants in our community |  |  |  |  |  |
| 3.9 | | I encourage to increase the no of elephants in the area |  |  |  |  |  |
| 3.10 | | Conservation of elephants can open a door for tourist attraction |  |  |  |  |  |
| 3.11 | | Elephants crop raiding doesn’t a significant issue in the area |  |  |  |  |  |
| 3.12 | | Elephants have the right to live in the area |  |  |  |  |  |
| 3.13 | | Elephants are important to the whole KSNP ecosystem |  |  |  |  |  |
| 3.14 | | Construction of water reservoirs assure elephant conservation |  |  |  |  |  |

**Note:** KSNP=Kafta Sheraro National Park

**4. Human-wildlife (elephant) interaction & communities’ perception about elephants**

4.1 Can you list the wildlife species observed in your locality?

4.2 Does wildlife cause problems in your locality?

No=0 Yes=1

4.3 If yes=1 for how long?

4.4 What are the problems of wildlife species in your areas?

| Conflict types | Response |
| --- | --- |
| 1.Crop damage (feeding and crash) |  |
| 2.Domestic animal injury (depredation) |  |
| 3.Disease transmission for animal |  |
| 4. Human injury |  |
| 5.Others (speacify) |  |

4.5 If your answer is crop damage (crop raiding), list the wildlife species that have brought problems in the past ten years and rank them based on the level given below.

| Name of wild animals | Rank (1=major; 2=moderate, 3=minor, 4=no problem) |
| --- | --- |
| 1. |  |
| 2. |  |
| 3. |  |
| 4. |  |
| 5. |  |

4.6 Did you have any problems with elephant crop raiding in 2018-2020?

No=0 Yes=1

4.7 Can you predict the trend of crop damage in the past ten year’s (10 years) period?

| Increased/increase | Decreased/decrease | Stay/stayed the same | Don’t know |
| --- | --- | --- | --- |
|  |  |  |  |

4.8 What are the crop types damaged by elephants?

| Crop type/ plant name | Season | Habitat | Time | Size (ha) | Rank |
| --- | --- | --- | --- | --- | --- |
| 1. |  |  |  |  |  |
| 2. |  |  |  |  |  |
| 3. |  |  |  |  |  |
| 4. |  |  |  |  |  |
| 5. |  |  |  |  |  |

4.9 Have you been forced to change your crops or abandon your farm because of elephant destruction?

No=0 Yes =1

4.10 Can you weight the level of crop damage impacts or how much crop has been damaged by elephant?

| High | Medium | Low | Don’t know (No complain) |
| --- | --- | --- | --- |
|  |  |  |  |

4.11 If your crop was severely damaged, was there any support or compensation given by the

Government / stakeholders?

No=0 Yes=1

4.12 In which season do you expect crop destruction to be more pronounced?

| Dry season | Wet season |
| --- | --- |
|  |  |

4.13Why are the elephant concentrated in your agricultural areas?

| 1.The farm is near to the park |  |
| --- | --- |
| 2.The farm is inside the park |  |
| 3.The farm is near to water point (riverside) |  |
| 4. Others (specify)………………………… |  |

4.14 What measures did you take to prevent elephant damage to crops?

| Method of protection | Response |
| --- | --- |
| 1. Gun sound and related noisy materials |  |
| 2. Local materials fences(physical barriers) |  |
| 3. Fire and flashlight |  |
| 4. Land use planning (alternative crop cultivation) |  |
| 5. No opinion about prevention |  |
| 5. Others (specify)………………………. |  |

4.15 What are the sustainable management strategies of elephant crop raiding you recommend?

| Sustainable strategies | Response |
| --- | --- |
| 1. Barriers |  |
| 2. Compensation |  |
| 3. Resettlement/ Relocate |  |
| 4. Traditional protection techniques |  |
| 5. Killing |  |
| 6. Others (specify)……………………. |  |

4.16 Do you expect Killing of elephants is a solution for crop damage?

No=0 Yes=1

4.17 How is your method of protection successful? Give your opinion.

| Effective | Moderate | Not effective | Don’t know |
| --- | --- | --- | --- |
|  |  |  |  |
